# Thrombin Generation Is Associated with Venous Thromboembolism Recurrence, but Not with Major Bleeding and Death in the Elderly: A Prospective Multicenter Cohort Study

**DOI:** 10.3390/jcm12186050

**Published:** 2023-09-19

**Authors:** Kristina Vrotniakaite-Bajerciene, Sereina Rütsche, Sara Calzavarini, Claudia Quarroz, Odile Stalder, Marie Mean, Marc Righini, Daniel Staub, Juerg H. Beer, Beat Frauchiger, Joseph Osterwalder, Nils Kucher, Christian M. Matter, Marc Husmann, Martin Banyai, Markus Aschwanden, Lucia Mazzolai, Olivier Hugli, Nicolas Rodondi, Drahomir Aujesky, Anne Angelillo-Scherrer

**Affiliations:** 1 Department of Hematology and Central Hematology Laboratory, Inselspital, Bern University Hospital, University of Bern, 3010 Bern, Switzerland; kristina.vrotniakaite-bajerciene@insel.ch (K.V.-B.); sereina.ruetsche@students.unibe.ch (S.R.); sara.calzavarini@gmail.com (S.C.); claudia.quarroz@bluewin.ch (C.Q.); 2 Department for BioMedical Research, University of Bern, 3010 Bern, Switzerland; 3 Clinical Trials Unit (CTU) Bern, University of Bern, 3010 Bern, Switzerland; odile.stalder@ctu.unibe.ch; 4 Department of General Internal Medicine, Inselspital, Bern University Hospital, University of Bern, 3010 Bern, Switzerland; marie.mean@chuv.ch (M.M.); nicolas.rodondi@insel.ch (N.R.); drahomirantonin.aujesky@insel.ch (D.A.); 5 Department of Medicine, Lausanne University Hospital, Lausanne University, 1005 Lausanne, Switzerland; 6 Division of Angiology and Hemostasis, Geneva University Hospital, 1205 Geneva, Switzerland; marc.righini@hcuge.ch; 7 Division of Angiology, Basel University Hospital, 4031 Basel, Switzerland; daniel.staub@usb.ch (D.S.); markus.aschwanden@usb.ch (M.A.); 8 Department of Internal Medicine, Cantonal Hospital of Baden, 5404 Baden, Switzerland; juerg-hans.beer@ksb.ch; 9 Department of Internal Medicine, Cantonal Hospital of Frauenfeld, 8501 Frauenfeld, Switzerland; beat.frauchiger@stgag.ch; 10 Cantonal Hospital of St. Gallen, 9000 St. Gallen, Switzerland; jo@j-osterwalder.ch; 11 Clinic of Angiology, University Hospital Zurich, 8091 Zurich, Switzerland; nils.kucher@usz.ch; 12 Department of Cardiology, University Heart Center, University Hospital Zurich, 8091 Zurich, Switzerland; christian.matter@uzh.ch; 13 Center for Translational and Experimental Cardiology (CTEC), Department of Cardiology, Zurich University Hospital and University of Zurich, 8091 Zurich, Switzerland; 14 Center for Vascular Diseases, Zurich-Stadelhofen, Stadelhoferstrasse 8, 8001 Zurich, Switzerland; marc.husmann@hin.ch; 15 Gefässpraxis Luzern Swiss AG, Pilatusstrasse 34, 6003 Lucerne, Switzerland; martin.banyai@spital-schwyz.ch; 16 Service of Angiology, Lausanne University Hospital, Lausanne University, 1005 Lausanne, Switzerland; lucia.mazzolai@chuv.ch; 17 Emergency Department, Lausanne University Hospital, Lausanne University, 1005 Lausanne, Switzerland; olivier.hugli@chuv.ch; 18 Institute of Primary Health Care (BIHAM), University of Bern, 3010 Bern, Switzerland

**Keywords:** thrombin generation, venous thromboembolism, elderly, bleeding, mortality

## Abstract

It is currently unknown whether thrombin generation is associated with venous thromboembolism (VTE) recurrence, major bleeding, or mortality in the elderly. Therefore, our aim was to prospectively study the association between thrombin generation and VTE recurrence, major bleeding, and mortality in elderly patients with acute VTE. Consecutive patients aged ≥65 years with acute VTE were followed for 2 years, starting from 1 year after the index VTE. Primary outcomes were VTE recurrence, major bleeding, and mortality. Thrombin generation was assessed in 551 patients 1 year after the index VTE. At this time, 59% of the patients were still anticoagulated. Thrombin generation was discriminatory for VTE recurrence, but not for major bleeding and mortality in non-anticoagulated patients. Moreover, peak ratio (adjusted subhazard ratio 4.09, 95% CI, 1.12–14.92) and normalized peak ratio (adjusted subhazard ratio 2.18, 95% CI, 1.28–3.73) in the presence/absence of thrombomodulin were associated with VTE recurrence, but not with major bleeding and mortality after adjustment for potential confounding factors. In elderly patients, thrombin generation was associated with VTE recurrence, but not with major bleeding and/or mortality. Therefore, our study suggests the potential usefulness of thrombin generation measurement after anticoagulation completion for VTE to help identify among elderly patients those at higher risk of VTE recurrence.

## 1. Introduction

Venous thromboembolism (VTE), comprising deep vein thrombosis (DVT) and pulmonary embolism (PE), constitutes a worldwide major health issue, and a leading cause of death [1]. VTE incidence increases with age due to the accumulation of risk factors and comorbidities predisposing to thrombosis [2,3,4,5]. The incidence rate is about 1/10,000 annually before age 40 years, rises after age 45, and approaches 5–6/1000 annually by age 80 [6]. In older patients, VTE results in higher mortality, but the rate of recurrence is no higher than in younger patients [3]. The morbidity burden of VTE on the older patient appears to be larger, with a higher increase in the incidence of PE compared with DVT with aging [6].

The recurrence rate of VTE is principally determined by the circumstances in which the index VTE occurs and varies between <3% and >8% annually after a first event [7]. The risk is greatest in patients whose first episode was associated with cancer, and lowest in those whose first episode was associated with a transient risk factor [7,8,9,10,11].

Because older patients are more likely to have comorbidities, they are not only at increased risk of VTE, but also of bleeding [5]. Therefore, managing anticoagulation in the elderly is often challenging. Since the risk of VTE recurrence is highest in the first 6 to 12 months after discontinuation of treatment for the initial event, and gradually decreases thereafter [3], the benefit of prolonged anticoagulation may be outweighed by the risk of clinically significant bleeding [12,13,14,15,16,17]. Consequently, the identification of older patients who might benefit from indefinite anticoagulant treatment is paramount. In order to facilitate the identification of these patients, the benefit/risk ratio should be carefully evaluated by considering clinical and laboratory information. Of these, D-dimer has been proposed over the past twenty years as one of the laboratory tests that can be used to evaluate the risk of VTE recurrence after cessation of anticoagulation [18,19,20]. Recently, a higher-than-expected recurrence rate has been observed in patients who discontinued anticoagulation in response to negative D-dimer results, particularly in men [21]. Another study showed that the long-term risk of recurrence in patients with a first unprovoked VTE and negative D-dimer results is not low enough to warrant discontinuation of anticoagulation in men, but can be envisaged in women [22]. Thus, the validation of an alternative test to D-dimer is justified.

Thrombin activity may be recorded by continuously measuring the cleavage of a fluorescent substrate, yielding a thrombin generation (TG) curve [23,24,25]. From this curve, several parameters can be extracted, including thrombin burst time, maximum amount of thrombin generated, TG rate, or total amount of thrombin generated. The TG assay proved to be a reliable predictor of recurrent VTE [26,27] and can therefore be used individually or in conjunction with D-dimer [28] to determine the risk of recurrence and the appropriate length of anticoagulation therapy. In a prospectively conducted cohort study including patients with a first unprovoked VTE, Hron et al. [29] reported that TG assessed after the cessation of anticoagulation can identify patients at a lower risk for recurrent VTE. In addition, a numerical simulation model showed that the TG assay was associated with the risk of first VTE [30]. TG increases with age [31,32] and TG parameters are associated with the risk of first venous thrombosis in older adults [33]. However, it is unknown if TG is associated with recurrent VTE, major bleeding, and mortality in the elderly.

Here, in a prospective cohort of VTE patients aged ≥65 years, we studied whether TG 1 year after index VTE is associated with VTE recurrence, major bleeding, and mortality for 2 years, starting from 1 year after the index VTE.

## 2. Materials and Methods

### 2.1. Cohort Sample

The study was performed between 09/2009 and 12/2013 as part of the SWIss venous Thromboembolism COhort (SWITCO65+), a multicenter prospective cohort study to evaluate medical outcomes and quality of life of elderly patients with acute symptomatic VTE at the 5 university hospitals and 4 non-university hospitals in Switzerland [34,35]. Consecutive patients aged ≥65 years with acute VTE were followed for 2 years, starting from 1 year after the index VTE. The patients had to give separate written consents for the clinical part of the study and for the future use of the blood samples. The study and biobank protocols were approved by the ethics committees of all participating hospitals [34,35]. The blood samples were collected between 09/2009 and 03/2012, and the patients were followed up until 12/2013. An outline of the study methods has been published [34,35].

### 2.2. Data Collection

For all registered patients, study nurses prospectively collected baseline information (Table 1). The follow-up consisted of one telephone interview and two face-to-face assessments during the first year of study participation, followed by semiannual contacts, alternating face-to-face assessments and telephone calls, and periodic revisions of the patient’s hospital chart. At each visit/contact, study nurses interviewed patients to receive information on the date and type of clinical events (recurrent VTE, bleeding, or death). If a clinical event had happened, this information was supplemented by reviewing medical records and interviewing patients’ primary care physicians and family members [35].

### 2.3. Blood Samples

Venous blood was collected into 0.106 M trisodium citrate S-Monovette (Sarstedt, Nümbrecht, Germany) one year after the index VTE. The samples were handled in accordance with the guidelines of the ISTH Scientific and Standardization Committee subcommittee [36,37]. The resulting platelet-free plasma was stored in small aliquots at −80 °C within one hour of blood collection [34].

### 2.4. Thrombin Generation Assay

TG measurements were performed 1 year after the index VTE with the calibrated automated thrombogram (CAT) assay (Stago, Asnières-sur-Seine, France) as previously described [38]. Two experimental settings were used.

In the first setting (referred later on as CAT_low tissue factor [TF]_), 74 μL PFP was added to 20 μL of a mixture of 1 pmol L^−1^ TF and 4 µmol L^−1^ phospholipids (PPP reagent LOW, Stago), and of recombinant human thrombomodulin (TM, Sekisui, Alveo AG, Switzerland) or 6 µL of HN-buffer (Hepes 20 mM, NaCl 140 mM, pH 7.4 + 5 mg mL^−1^ BSA), in a 96-well round bottom microtiter plate (Immulon2HB, Thermo Fischer Scientific, Reinach, Switzerland). The concentration of TM was tested in a preliminary assay and selected by the ability to decrease by 50% the peak of thrombin.

For the second setting (referred later on as CAT_high TF_), 74 μL PFP was added to 20 μL of a mixture of TF and phospholipids (7:3 mixture PPP reagent HIGH and MP reagent, Stago, Asnières-sur-Seine, France) and 6 µL of recombinant human activated protein C (APC) (Enzyme Research, Swansea, United Kingdom) or HN-buffer, in a 96-well round bottom microtiter plate. The concentration of APC was tested in a preliminary assay and selected by the ability to decrease by 90% the endogenous thrombin potential (ETP) [31].

The reaction was initiated with 20 μL of a mixture of fluorogenic substrate and CaCl_2_ (Fluobuffer, Stago, Asnières-sur-Seine, France) and fluorescence was measured using a fluorescence plate reader (Fluoroskan Ascent, Thermo Labsystems, Helsinki, Finland). All experiences were carried out in duplicate at 37 °C for each assay. In addition, the same reference plasma (Cryocheck Reference Control Normal, Precision Biologic, Dartmouth, Canada) was tested in all experiments in order to correct day-to-day variations. TG curves were generated using the Thrombinoscope software version 5.0.0.742 (Thrombinoscope BV, Maastricht, The Netherlands). Lag time, peak height, time to peak, ETP, and the ETP ratio obtained in the presence/absence of TM or APC was calculated. Results from the reference plasma were used to calculate the normalized peak ratio as follows [23,39]: (Patient peak_+TM_/Patient peak_-TM_)/(Reference peak_+TM_/Reference peak_-TM_) and (Patient peak_+APC_/Patient peak_-APC_)/(Reference peak_+APC_/Reference peak_-APC_). Results from the control plasma were also used to calculate the normalized ETP ratio as follows [23,39]: (Patient ETP_+TM_/Patient ETP_-TM_)/(Reference ETP_+TM_/Reference ETP_-TM_) and (Patient ETP_+APC_/Patient ETP_-APC_)/(Reference ETP_+APC_/Reference ETP_-APC_).

For all measured parameters, intra-assay coefficients of variation (CV) were <10%, and inter-assay CV, <15%.

### 2.5. Outcome Variables

The primary outcomes of the study were symptomatic VTE recurrence, major bleeding, and overall mortality between 1 year and 3 years after the index VTE. We adjudicated outcomes by interviewing the patient or patient representative, interviewing the patient’s treating physician, and reviewing hospital records [35].

Recurrent VTE was defined as fatal or new nonfatal PE or new DVT [40]. The diagnosis of recurrent VTE during follow-up was made based on the following criteria: for DVT, based on abnormal ultrasound findings; and for PE, based on CT or angiography displaying new intraluminal defects, or based on ventilation–perfusion lung scans exhibiting a high-probability pattern with new perfusion defects. A new proximal DVT, based on abnormal ultrasound findings, associated with one or more new PE symptom(s) was also regarded as recurrent PE.

Major bleeding was defined as fatal bleeding, symptomatic bleeding at critical sites, or clinically overt bleeding accompanied by a decrease in hemoglobin level of at least 20 g L^−1^, or resulting in the transfusion of two or more units of packed red blood cells [41].

A committee of three blinded clinical experts validated all outcomes and classified the deaths as definitely due to PE, possible PE, major bleeding, or other causes [35]. The definitive classification was conducted based on the total consensus of this committee [35].

### 2.6. Statistical Analysis

Patient characteristics were compared between groups using the Chi-squared test for categorical variables and the non-parametric Mann–Whitney U test for continuous variables.

We calculated the incidence rates of a first VTE recurrence, first major bleed, or death at 2 years, starting 1 year after the index VTE, by the level of the different TG parameters separately for anticoagulated and non-anticoagulated patients.

We performed a complete case analysis for the different TG parameters, only patients with available values were analyzed.

The discriminative power of peak height or ETP in predicting VTE recurrence, major bleeding, and mortality was determined by calculating the Harrell’s C concordance statistic. Associations between TG parameters, analyzed as continuous variables and the time to the first VTE recurrence and major bleeding, were assessed by the use of competing risk regression accounting for non-PE-related and non-bleeding-related death, respectively, as a competing event [42]. The method yields subhazard ratios (SHR) with their corresponding 95% confidence intervals (CI). For mortality, an ordinary Cox regression with robust standard errors was calculated. We adjusted the model for previously published predictors of VTE recurrence or major bleeding [40,41,43,44,45,46,47,48,49,50,51,52]. For overall mortality, analyses were adjusted for age, gender, cancer, provoked VTE, prior VTE, overt PE, renal disease, history of major bleeding, heart failure, chronic lung disease, elevated heart rate, low blood pressure, low oxygen, and periods of anticoagulation as a time-varying covariate [49,53]. The missing values in the adjustment variables were imputed to have a normal or an absence status.

## 3. Results

### 3.1. Study Sample

**Figure 1:**
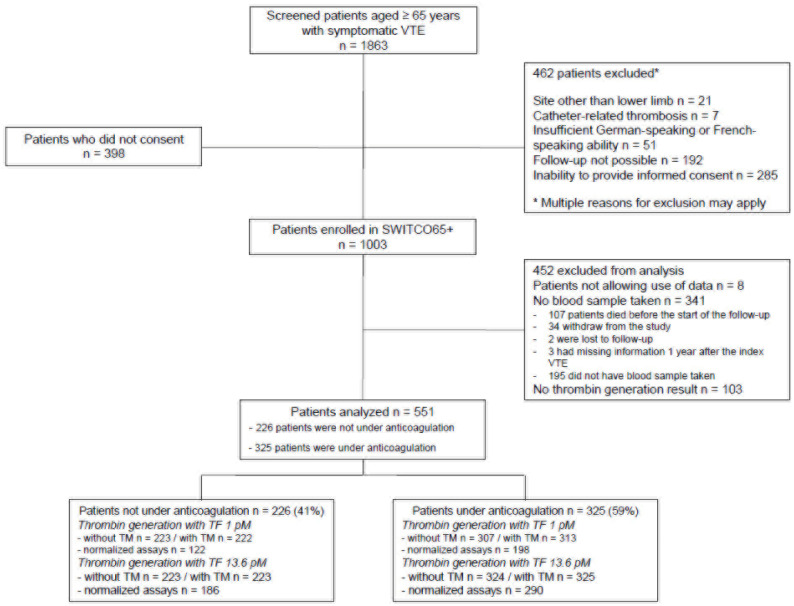
Flow diagram of patients included in the study. APC, activated protein C; TF, tissue factor; TM, thrombomodulin; VTE, venous thromboembolism.

A total of 1863 were screened. We excluded 462 who had at least one of the following exclusion criteria: thrombosis at a different site than the lower limb (n = 21), catheter-related thrombosis (n = 7), insufficient ability to speak German or French (n = 51), follow-up not possible (n = 192), inability to provide informed consent (n = 285), leaving a sample of 1401 eligible patients. After the exclusion of 398 patients who refused to provide informed consent, our initial study sample comprised 1003 patients (54% of screened patients) [35]. Of the 1003 enrolled patients aged ≥65 years with acute VTE, we excluded 452 patients, yielding a study sample of 551 patients (Figure 1). Of 452 excluded patients, 341 patients had no blood analyses (including TG) and 103 had blood analyses, but no validated TG results 1 year after the index VTE. Characteristics of patients with results of TG with/without TM one year after the index VTE are shown in Table S2, and those of patients with results of normalized assays with/without TM, in Table S3.

Patient characteristics of the study cohort are reported in Table 1. Overall, 232 patients (42%) were females, and the median age was 74 years (interquartile range (IQR) 69–79 years). All patients but one were Caucasians. Three hundred and nine patients (56%) presented with an index PE.

Sixty patients (11%) had cancer-related VTE, 113 (21%) had provoked VTE, and 378 (69%) had an unprovoked index VTE. One hundred and sixty-eight patients (30%) had experienced prior VTE. Patients still under anticoagulation 1 year after the index VTE were more inclined to have PE only as index VTE, and, if DVT was the index VTE, they were more likely to have a proximal DVT. They were also more prone to have unprovoked VTE. Finally, these patients were also less immobilized and had more major surgery during the last 3 months. They were more likely to have heart failure. Characteristics of tested and non-tested patients for TG are provided in Table S1. Untested patients were slightly older, more immobilized during the last 3 months, and displayed more major bleeding and anemia, and cancer-associated VTE than tested patients.

### 3.2. Thrombin Generation Parameters in Study Samples

TG was assessed in 551 patients 1 year after the index VTE (Figures S1 and S2). The settings of TG assays and subgroups of patients studied for TM, APC resistance, and normalized assays are summarized in Figure 1. Among the 551 studied patients, 226 were not under anticoagulation and 325 patients were under anticoagulation (Table 1 and Figure 1).

**Figure 2:**
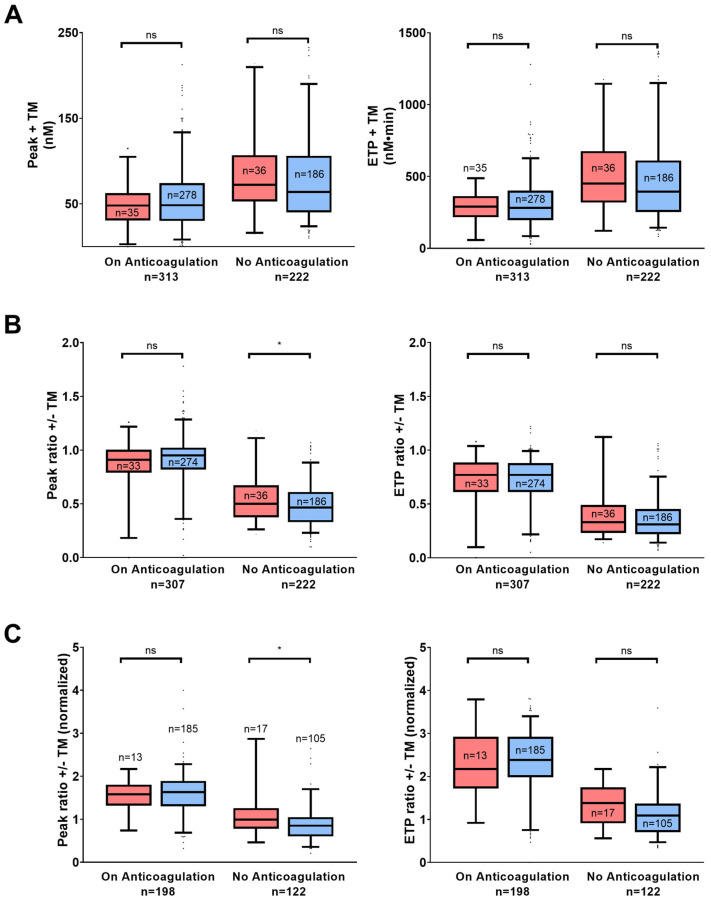
Thrombin generation parameters in patients under anticoagulation and not under anticoagulation 12 months after the index venous thromboembolism (VTE) at 1 pM TF with and without thrombomodulin (TM). (**A**), Peak and endogenous thrombin potential (ETP) with TM. (**B**), Peak and ETP ratio with/without TM. (**C**), Peak and ETP ratio with/without TM normalized with reference plasma. The red boxes indicate patients with VTE recurrence and the blue boxes, those without VTE recurrence up to 24 months following the index VTE. Box plots of thrombin generation parameters are presented as median with interquartile range (5–95%). Groups were compared using Mann–Whitney U test. ns, not significant; *, *p* < 0.05.

TG parameters with 1 pM TF in the absence of TM were comparable in patients with and without recurrent VTE in both anticoagulated and non-anticoagulated patients (Figure S3). However, both peak ratio (patients with VTE recurrence, median 0.54, IQR 0.26–1.11 versus patients without VTE recurrence, median 0.46, IQR 0.23–0.88, *p* < 0.05) and normalized peak ratio (patients with VTE recurrence, median 0.99, IQR 0.46–2.87 versus patients without VTE recurrence, median 0.85, IQR 0.36–1.70, *p* < 0.05) with/without TM were slightly but significantly higher in patients with recurrent VTE and not under anticoagulation (*p* < 0.05) (Figure 2). When TG was measured with 13.6 pM TF, only time to peak was prolonged in the group of patients not under anticoagulation and with recurrent VTE (Figures S4 and S5).

**Figure 3:**
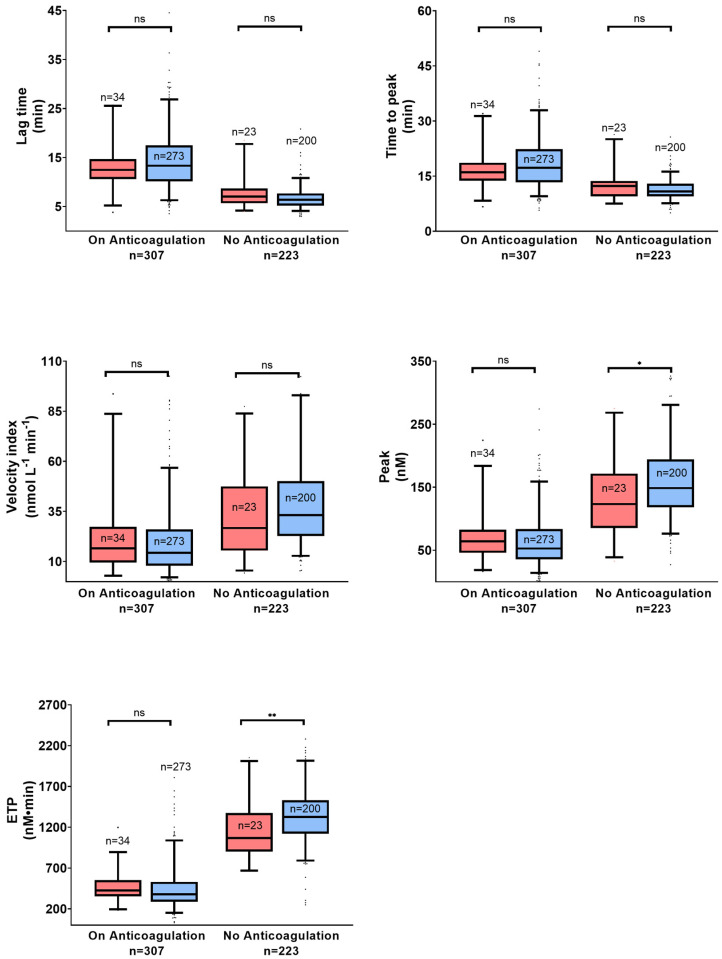
Thrombin generation parameters in patients under anticoagulation and not under anticoagulation 12 months after index venous thromboembolism (VTE) at 1 pM TF without thrombomodulin (TM). The red boxes indicate patients who had a major bleeding event and the blue boxes, those without major bleeding up to 24 months following the index VTE. Box plots of thrombin generation parameters are presented as median with interquartile range (5–95%) as indicated. Groups were compared using the Mann–Whitney U test. ETP, endogenous potential; ns, not significant; * *p* < 0.05; ** *p* < 0.01.

Peak (patients with major bleeding, median 123.2 nM, IQR 38.8–268.3 versus patients without major bleeding, median 148.6 nM, IQR 76.3–280.5, *p* < 0.05) and ETP (patients with major bleeding, median 1068 nM.min, IQR 669–2012 versus patients without major bleeding, median 1325 nM·min, IQR 791–2015, *p* < 0.05) measured using 1 pM TF were lower in non-anticoagulated patients who presented major bleeding during the follow-up period (Figure 3). However, peak and ETP in the presence of TM with 1 pM TF were higher in anticoagulated patients who had major bleeding during follow-up (Figure S6). There was no difference in thrombin generation parameters measured in the presence/absence of APC with 13.6 pM TF between anticoagulated and non-anticoagulated patients (Figures S7 and S8).

Lag time and time to peak, in the absence of TM and APC and with 1 pM or 13.6 pM TF in non-anticoagulated patients who died during follow-up, were longer than in patients who did not die during this period (Figures S9–S11). In addition, ETP with APC as well as normalized and non-normalized ETP ratio with/without APC using 13.6 pM TF were lower in non-anticoagulated patients who died than in non-anticoagulated patients who did not die during the follow-up period (Figure S12).

### 3.3. Incidence Rates of VTE Recurrence, Major Bleeding, and Mortality, and Thrombin Generation

**Figure 4:**
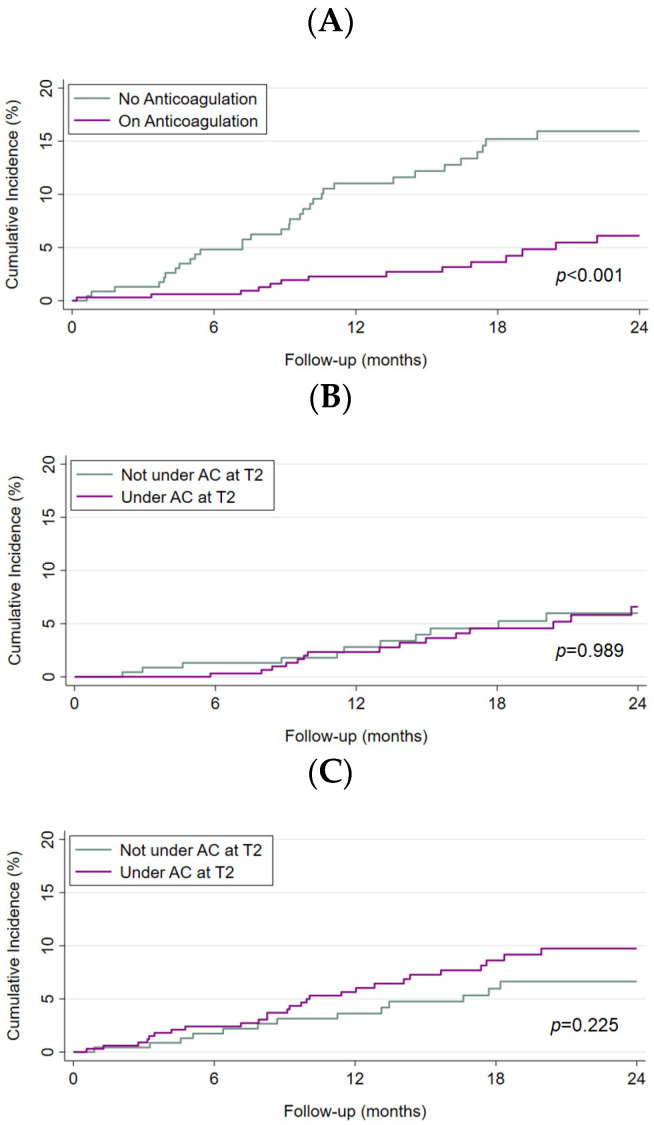
Cumulative incidence rates of venous thromboembolism (VTE) recurrence, major bleeding, and mortality in patients under anticoagulation versus not under anticoagulation 1 year after the index VTE. The cumulative incidence rates of VTE recurrence (**A**), major bleeding (**B**), and mortality (**C**) were estimated with the Kaplan–Meier method, and survivor functions across groups were compared by use of the log-rank test. AC, anticoagulation, T2, 1 year after the index VTE.

After a follow-up 2 years, starting 1 year after the index VTE, 46 patients had developed recurrent VTE, resulting in an incidence rate of 5.5 events per 100 person-years (95% CI 4.2–7.4), and 26 patients presented with major bleeding (incidence rate: 3.1 events per 100 person-years (95% CI 2.1–4.5). During the same period, 40 of 863 patients had died (mortality rate of 4.6 events per 100 person-years; 95% CI 3.4–6.3). The 2-year cumulative incidence of VTE recurrence was lower in anticoagulated patients 12 months after the index VTE (Figure 4A). In contrast, the 2-year cumulative incidence of major bleeding and mortality was comparable in both groups (Figure 4B,C).

During the 2-year follow-up, incidence rates (IR) of VTE recurrence and major bleeding were higher in non-anticoagulated patients with either peak ratio or normalized peak ratio, or ETP ratio or normalized ETP ratio with/without TM values above median than in those with peak and ETP ratio below the median (Table 2). In patients under anticoagulation, the IR of VTE recurrence during the 2-year follow-up was lower than in non-anticoagulated patients (Table S4).

The mortality rate was higher in non-anticoagulated patients with peak ratio or normalized peak ratio, or high ETP ratio with/without TM than in those with peak and ETP ratio below median (Table 2). However, it was comparable in non-anticoagulated patients with low and high normalized ETP ratios in the presence or absence of TM (Table 2).

### 3.4. Discriminative Power of Thrombin Generation Parameters for Outcomes

To assess the discriminative power of TG parameters, *C*-statistic values (95% CI) were calculated for TG parameters where TM was involved in non-anticoagulated patients 1 year after index VTE (Table 3). Peak ratio with/without TM (*C*-statistic 0.70, 95% CI [0.59 to 0.81]) and ETP with/without TM (*C*-statistic 0.70, 95% CI [0.60 to 0.80]) normalized with reference plasma were discriminatory for VTE recurrence, but not for major bleeding and overall mortality from 1 to 3 years following index VTE. (Table 3). In patients under coagulation, only peak ratio with/without TM (*C*-statistic 0.74, 95% CI [0.60 to 0.88]) was discriminatory for VTE recurrence, but not for major bleeding and overall mortality from 1 to 3 years following index VTE (Table S5).

### 3.5. Association between Thrombin Generation Parameters and Outcomes

We investigated the association of various TG parameters measured in non-anticoagulated patients (Table 4) and in patients under anticoagulation (Table S6) 1 year after the index VTE with VTE recurrence, major bleeding, and mortality from 1 to 3 years following the index VTE.

Peak ratio in the presence/absence of TM was associated with VTE recurrence (SHR: 4.09, 95% CI [1.12–14.92] after adjustment for potential confounding factors for the risk of VTE recurrence). This association remained when peak ratio was normalized with reference plasma (SHR: 2.18, 95% CI [1.28–3.73] after adjustment for potential confounding factors for the risk of VTE recurrence). However, peak ratio in the presence/absence of TM was not associated with major bleeding and overall mortality. ETP ratio in presence/absence along with ETP ratio with/without APC normalized with reference plasma was not associated with VTE recurrence, major bleeding, or mortality. In patients under anticoagulation, peak ratio in presence/absence of TM was associated with VTE recurrence (SHR: 0.11, 95% CI [0.03–0.45] after adjustment for potential confounding factors for the risk of VTE recurrence) (Table S6). However, peak ratio in the presence/absence of TM was not associated with major bleeding and overall mortality.

## 4. Discussion

We prospectively followed 551 elderly patients for 2 years, starting from 1 year after the index VTE. Of these, 59% were still anticoagulated 1 year after the index VTE. Anticoagulated patients were more likely to experience prior VTE and unprovoked VTE than patients who were no longer anticoagulated 1 year after the initial VTE (prior VTE: 43% versus 13%; unprovoked: VTE 76% versus 58%). As expected, patients still under anticoagulation 1 year after the index VTE were less likely to develop recurrent VTE in the next 2 years than patients without anticoagulation, while the incidence of major bleeding and mortality did not differ between the two patient groups. This observation may indicate that selected elderly patients may benefit from extended anticoagulation without having a higher risk of major bleeding or death.

In this cohort, we measured thrombin generation at 1 pM TF in the absence/presence of soluble TM, and at 13.6 pM TF in the absence/presence of APC. Thrombin generation curves at 13.6 TF were faster and higher than those obtained at 1 pM TF either in anticoagulated or non-anticoagulated patients, as expected [31]. Moreover, thrombin generation curves either at 1 pM TF or 13.6 pM TF were slower and lower in anticoagulated than in non-anticoagulated patients as expected [31].

We observed that several TG parameters were not only different for primary outcomes, but also discriminatory for VTE recurrence in non-anticoagulated patients and associated with it after adjustment for potential confounding factors.

TG has been used alone [26,27] or in conjunction with D-dimer [28] to evaluate the recurrence of VTE and to determine the length of secondary thromboprophylaxis. Several aspects of the design of our study differ from those of previous ones involving TG measurement to assess VTE recurrence [26,27,28,29,70], namely: (1) TG measured 1 year after the index VTE; (2) TG assessed in both anticoagulated and non-anticoagulated patients; (3) 2-year follow-up, starting 1 year after the initial VTE; (4) inclusion of patients with provoked and cancer-related VTE; and (5) cohort of elderly patients.

Our data showed that both peak ratio and peak ratio normalized with reference plasma with/without TM were higher in non-anticoagulated patients with recurrent VTE than in those without recurrent VTE. Time to peak at 13.6 pM TF was prolonged in non-anticoagulated patients with recurrent VTE. Peak along with ETP ratio with/without TM showed a trend for a discriminatory power for VTE recurrence, whereas both peak and ETP ratio with/without TM normalized with reference plasma showed a significant discriminatory power for VTE recurrence. These parameters did not display a discriminatory power for both major bleeding and mortality. In addition, peak ratio in the presence/absence of TM was associated with VTE recurrence after adjustment for potential confounding factors for the risk of VTE recurrence. This association remained when peak ratio was normalized with reference plasma. However, peak ratio in the presence/absence of TM was not associated with major bleeding and overall mortality. In patients under anticoagulation, peak with/without TM showed a significant discriminatory power for VTE recurrence, but not for major bleeding and mortality. Similarly, peak ratio in the presence/absence of TM was associated with VTE recurrence after adjustment for potential confounding factors for the risk of VTE recurrence. However, peak ratio in the presence/absence of TM was not associated with major bleeding and overall mortality. To our knowledge, the association between TG parameters and VTE recurrence has not been previously demonstrated in the elderly. Notably, normalized peak and ETP ratios were more discriminatory than those that were not normalized in non-anticoagulated patients, demonstrating the importance of the use of a reference plasma for TG measurement in these patients.

Non-anticoagulated patients who developed a major bleeding event during the follow-up period had a lower peak and ETP at 1 pM TF than those who did not. Further validation of this parameter may help identify an elderly population, in whom an extension of the anticoagulation might be potentially harmful. Peak and ETP with TM were higher in anticoagulated patients who had a major bleeding event than in those who did not, pointing to the potential usefulness of TG in monitoring elderly patients under anticoagulation. Importantly, TG measurement has not been previously reported for major bleeding assessment in this age group with VTE. However, we were unable to demonstrate an association between TG parameters and major bleeding.

Lag time and time to peak with 1 and 13.6 pM TF were longer, and ETP measured with APC and ETP ratios obtained in the presence/absence of APC with 13.6 pM TF were lower in non-anticoagulated patients who died during the follow-up than in those who did not. However, normalized ETP ratio obtained in the presence/absence of APC was not associated with overall mortality. A few studies have examined the association between TG and mortality. The PROSPER study, which enrolled only older adults, demonstrated positive associations of vascular mortality with lag time and peak height and of total mortality with lag time [71]. After adjustment for interleukin-6 and C-reactive protein levels, however, the associations were no longer statistically significant, pointing to inflammation as a contributor to increased TG in this population. A smaller study showed that increased ETP and peak height (with 5 pM TF), independent of age, sex, and cardiovascular risk factors, were associated with an enhanced risk of cardiovascular death in patients with acute coronary syndrome [72]. In a recent large adult population-based study, an association was found between lag time with 1 pM TF and overall mortality and a relation, between elevated ETP with 5 pM TF and increased risk of death [73]. In another study, it was shown that age-dependent TG predicts VTE occurrence and mortality within 30 days after multiple trauma [74]. Although our study showed no significant differences and associations in peak or ETP regarding overall mortality, a significantly higher APC resistance in non-anticoagulated alive patients shows a promising further research topic. Tissue factor pathway inhibitor is found to be one of the major determinants to prolong lag time and reduce ETP in the presence of APC and ETP ratios with/without APC lowering the APC resistance in TG [31], and it is known to be elevated in presence of co-morbidities such as obesity and diabetes mellitus [75], which may lead to a higher mortality rate. However, our findings cannot be explained by this fact entirely as the distribution of these co-morbidities was comparable in both alive and dead non-anticoagulated patients of this cohort. Hence, another determinant of this study’s finding should be considered.

Our study has some limitations. First, the study included 565 patients and 59% of them were still anticoagulated at the time of TG measurement. Second, TM and APC resistance assays as well as normalized TG were only performed in a subset of patients. The reason for creating this subgroup of patients was that there were not enough plasma materials for a complete analysis in some patients. This is because the initial analysis planned only TG testing without TM resistance and normalized TG assays. Third, all the patients but one were Caucasians; therefore, our findings may not be extended to other ethnicities. Fourth, a significant number of patients had comorbidities including cancer (11%). Therefore, mortality from comorbid disease tends to be higher than the rate of VTE recurrence, because patients with limited life expectancy often do not have time to develop recurrent VTE. Fifth, the treatment of VTE has evolved since the establishment of this cohort: direct oral anticoagulants are used instead of VKAs for the vast majority of patients. Therefore, it is not clear whether the findings can be generalized to patients treated with direct oral anticoagulants. Sixth, protein C and S could not be measured in patients under anticoagulation, because most of them were receiving VKA.

In conclusion, we demonstrated that several TG parameters were discriminatory for VTE recurrence in non-anticoagulated elderly patients and were associated with them, but not for the other primary outcomes (major bleeding and overall mortality). Therefore, our study suggests the potential usefulness of TG measurement after anticoagulation completion for VTE to help identify among elderly patients those at higher risk of VTE recurrence. The addition of TG testing may also help to improve the performance of validated assessment measures of the risk of thrombosis. These findings will set the basis for a larger prospective study.

## Figures and Tables

**Table 1:** Patient characteristics by anticoagulation status one year after index venous thromboembolism (VTE).

Characteristic	All n (%) or Median (IQ-Range)	Not under Anticoagulation One Year after Index VTE n (%) or Median (IQ-Range)	Under Anticoagulation One Year after Index VTE n (%) or Median (IQ-Range)	*p*-Value
Total number of patients	551	226	325	
Patient age (years)	74.0 (69.0;79.0)	74.0 (68.0;78.0)	75.0 (69.0;80.0)	0.321
Female sex ^1^	232 (42)	105 (46)	127 (39)	0.096
Patient race				
Caucasian	550 (100)	225 (100)	325 (100)	
African	1 (0)	1 (0)	0 (0)	
Index VTE event				<0.001
PE only	309 (56)	108 (48)	201 (62)	
DVT only	170 (31)	96 (42)	74 (23)	
PE and DVT	72 (13)	22 (10)	50 (15)	
Index DVT type ^2^				<0.001
proximal DVT only	99 (18)	46 (20)	53 (16)	
distal DVT only	56 (10)	40 (18)	16 (5)	
proximal and distal DVT	87 (16)	32 (14)	55 (17)	
Type of index VTE				<0.001
cancer-related VTE	60 (11)	27 (12)	33 (11)	
provoked index VTE	113 (21)	68 (30)	45 (14)	
unprovoked index VTE	378 (69)	131 (58)	247 (76)	
Current estrogen therapy during the last 3 months	18 (3)	9 (4)	9 (3)	0.471
Immobilization during the last 3 months	95 (17)	59 (26)	36 (11)	<0.001
Major surgery during the last 3 months	80 (15)	52 (23)	28 (9)	<0.001
Prior VTE	168 (30)	29 (13)	139 (43)	<0.001
PTS ^2^	291 (53)	121 (54)	170 (52)	0.661
History of major bleeding ^2^	43 (8)	18 (8)	25 (8)	1.000
Chronic liver disease	8 (1)	4 (2)	4 (1)	0.722
Chronic renal disease	98 (18)	34 (15)	64 (20)	0.175
Chronic or acute heart failure	65 (12)	17 (8)	48 (15)	0.010
Anemia ^2^	181 (33)	84 (37)	97 (30)	0.023
Concomitant antiplatelet therapy	174 (32)	64 (28)	110 (34)	0.192
Concomitant antiplatelet/NSAID therapy	204 (37)	75 (33)	129 (40)	0.128
Heart rate of ≥110 beats min^−1^ ^2^	46 (8)	15 (7)	31 (10)	0.275
Systolic BP of <100 mmHg ^2^	11 (2)	3 (1)	8 (2)	0.538
Arterial oxygen saturation of <90% ^2^	51 (9)	15 (7)	36 (11)	0.280
D-dimer at the time of the index VTE ^2^	2456 (1598;3746.5)	2404 (1681;3766)	2488 (1531;3705)	0.899
D-dimer 1 year after the index VTE ^2^	627 (386;1119)	940 (565;1528)	503 (322;787)	<0.001
Overall anticoagulation duration (days)	668 (213;979)	191 (145.5;283)	900 (710;1225.5)	<0.001
Anticoagulation duration until 1 year after the index VTE (days)	353 (194;365)	185.5 (120.8;210.8)	363 (357;371)	<0.001
Anticoagulation duration from 1 year after the index VTE (days)	344 (0;693)	0 (0;0)	535 (352.5; 871)	<0.001

Abbreviations: BP, blood pressure; BMI, body mass index; DVT, deep vein thrombosis; IQR, interquartile range; NSAID, non-steroidal anti-inflammatory drug; PE, pulmonary embolism; PTS, post-thrombotic syndrome. ^1^ Assigned at birth; ^2^ values were missing for presence of index DVT type (56%), PTS (2%), anemia (8%), heart rate of ≥110 beats min^−1^ (3%), systolic BP of <100 mmHg (2%), arterial oxygen saturation of <90% (23%), D-dimer at the time of the index VTE (8%), D-dimer 12 months after the index VTE (1%).

**Table 2:** Incidence rate of venous thromboembolism (VTE) recurrence, major bleeding, or overall mortality per 100 person-years—from 1 to 3 years after index VTE in non-anticoagulated patients.

		No of Patients	No of Events/Person-Years	Incidence Rate (95%-CI)
Peak ratio obtained in presence/absence of TM				
VTE recurrence				
	All	222	32/322.3	9.9 (7.0 to 14.0)
	≤median	111	12/172.0	7.0 (4.0 to 12.3)
	>median	111	20/150.3	13.3 (8.6 to 20.6)
Major bleeding				
	All	222	11/342.2	3.2 (1.8 to 5.8)
	≤median	111	2/182.0	1.1 (0.3 to 4.4)
	>median	111	9/160.3	5.6 (2.9 to 10.8)
Overall mortality				
	All	222	13/348.5	3.7 (2.2 to 6.4)
	≤median	111	4/182.8	2.2 (0.8 to 5.8)
	>median	111	9/165.8	5.4 (2.8 to 10.4)
Normalized peak ratio in presence/absence of TM				
VTE recurrence				
	All	122	17/156.1	10.9 (6.8 to 17.5)
	≤median	61	4/89.1	4.5 (1.7 to 12.0)
	>median	61	13/67.0	19.4 (11.3 to 33.4)
Major bleeding				
	All	122	3/165.6	1.8 (0.6 to 5.6)
	≤median	61	0/91.2	0.0 (−)
	>median	61	3/74.4	4.0 (1.3 to 12.5)
Overall mortality				
	All	122	4/167.0	2.4 (0.9 to 6.4)
	≤median	61	1/91.2	1.1 (0.2 to 7.8)
	>median	61	3/75.8	4.0 (1.3 to 12.3)
ETP ratio obtained in presence/absence of TM				
VTE recurrence				
	All	221	31/321.5	9.6 (6.8 to 13.7)
	≤median	111	12/174.5	6.9 (3.9 to 12.1)
	>median	110	19/147.0	12.9 (8.2 to 20.3)
Major bleeding				
	All	221	11/340.2	3.2 (1.8 to 5.8)
	≤median	111	4/182.9	2.2 (0.8 to 5.8)
	>median	110	7/157.4	4.4 (2.1 to 9.3)
Overall mortality				
	All	221	13/346	3.8 (2.2 to 6.5)
	≤median	111	5/185.5	2.7 (1.1 to 6.5)
	>median	110	8/161.0	5.0 (2.5 to 9.9)
Normalized ETP ratio obtained in presence/absence of TM				
VTE recurrence				
	All	122	17/156.1	10.9 (6.8 to 17.5)
	≤median	61	6/84.9	7.1 (3.2 to 15.7)
	>median	61	11/71.2	15.5 (8.6 to 27.9)
Major bleeding				
	All	122	3/165.6	1.8 (0.6 to 5.6)
	≤median	61	1/87.8	1.1 (0.2 to 8.1)
	>median	61	2/77.8	2.6 (0.6 to 10.3)
Overall mortality				
	All	122	4/167.0	2.4 (0.9 to 6.4)
	≤median	61	2/87.8	2.3 (0.6 to 9.1)
	>median	61	2/79.1	2.5 (0.6 to 10.1)

All experiments were conducted in the presence of 1 pM tissue factor.

**Table 3:** Discriminative power of thrombin generation parameters involving thrombomodulin (TM) for outcomes—from 1 to 3 years following the index venous thromboembolism (VTE) in not anticoagulated patients.

Thrombin Generation Parameters Measured One Year after the Index VTE	No. of Events/no. of Patients	*C*-Statistics (95% Confidence Interval)
Peak ratio obtained in presence/absence of TM		
VTE recurrence	32/222	0.60 (0.51 to 0.69)
Major bleeding	11/222	0.65 (0.50 to 0.80)
Overall mortality	13/222	0.59 (0.45 to 0.73)
Normalized peak ratio obtained in presence/absence of TM		
VTE recurrence	17/122	0.70 (0.59 to 0.81)
Major bleeding	3/122	0.65 (0.55 to 0.75)
Overall mortality	4/122	0.63 (0.36 to 0.89)
ETP ratio obtained in presence/absence of TM		
VTE recurrence	31/221	0.59 (0.50 to 0.69)
Major bleeding	11/221	0.63 (0.48 to 0.77)
Overall mortality	13/221	0.44 (0.32 to 0.56)
Normalized ETP ratio obtained in presence/absence of TM		
VTE recurrence	17/122	0.70 (0.60 to 0.80)
Major bleeding	3/122	0.48 (0.37 to 0.58)
Overall mortality	4/122	0.66 (0.46 to 0.87)

All experiments were conducted in the presence of 1 pM tissue factor (TF).

**Table 4:** Association between thrombin generation parameters and venous thromboembolism (VTE) recurrence, major bleeding, and overall mortality—from 1 to 3 years following the index VTE in not anticoagulated patients.

	n/N (%)	Crude Subhazard Ratio (95% Confidence Interval)	Adjusted Subhazard Ratio (95% Confidence Interval)
Peak ratio obtained in presence/absence of TM (TF 1 pM)			
VTE recurrence	32/222 (14.4)	3.94 (1.00 to 15.49)	4.09 (1.12 to 14.92)
Major bleeding	11/222 (5.0)	5.01 (0.67 to 37.24)	5.65 (0.83 to 38.71)
Overall mortality	13/222 (5.9)	1.89 (0.33 to 10.75)	2.93 (0.39 to 21.71)
Normalized peak ratio obtained in presence/absence of TM (TF 1 pM)			
VTE recurrence	17/122 (13.9)	2.21 (1.30 to 3.77)	2.18 (1.28 to 3.73)
Major bleeding	3/122 (2.5)	1.35 (0.84 to 2.18)	-
Overall mortality	4/122 (3.3)	1.36 (0.50 to 3.67)	-
ETP ratio obtained in presence/absence of TM (TF 1 pM)			
VTE recurrence	31/221 (14.0)	3.10 (0.86 to 11.24)	2.88 (0.82 to 10.09)
Major bleeding	11/221 (5.0)	3.38 (0.40 to 28.79)	3.02 (0.38 to 23.97)
Overall mortality	13/221 (5.9)	0.80 (0.16 to 3.97)	0.80 (0.10 to 6.55)
Normalized ETP ratio obtained in presence/absence of APC (TF 13.6 pM)			
VTE recurrence	17/122 (13.9)	1.82 (1.01 to 3.29)	1.80 (0.99 to 3.27)
Major bleeding	3/122 (2.5)	0.81 (0.54 to 1.23)	-
Overall mortality	4/122 (3.3)	1.58 (0.71 to 3.50)	-

Abbreviations: APC, activated protein C; ETP, endogenous thrombin potential; TM, thrombomodulin. Adjustments: VTE recurrence was adjusted for age, cancer, provoked VTE, prior VTE, overt pulmonary embolism, renal disease, and periods of anticoagulation (oral or parenteral anticoagulation) as a time-varying covariable [43,44,45,46,47,48,49,50,51,52]. Major bleeding was adjusted for age, cancer, provoked VTE, prior VTE, overt pulmonary embolism, renal disease, history of major bleeding, anemia, antiplatelet therapy, and periods of anticoagulation as time-varying covariate [54,55,56,57,58,59,60,61,62,63,64,65,66,67,68,69]. Mortality was adjusted for age, gender, cancer, provoked VTE, prior VTE, overt pulmonary embolism, renal disease, history of major bleeding, heart failure, chronic lung disease, high pulse, low blood pressure, low oxygen, and periods of anticoagulation as a time-varying covariate [49,53].

## Data Availability

There were no publicly archived datasets analyzed or generated during the study.

## Supplementary Materials

The following supporting information can be downloaded at: https://www.mdpi.com/article/10.3390/jcm12186050/s1, Table S1. Patient characteristics by thrombin generation testing status one year after index venous thromboembolism (VTE), Table S2. Characteristics of patients with results of thrombin generation with/without thrombomodulin one year after index venous thromboembolism (VTE), Table S3. Characteristics of patients with results of normalized assays with/without thrombomodulin one year after index venous thromboembolism (VTE), Table S4. Incidence rate of venous thromboembolism (VTE) recurrence, major bleeding, or overall mortality per 100 person-years—from 12 months until 36 months after index VTE in anticoagulated patients, Table S5. Discriminative power of thrombin generation parameters involving thrombomodulin (TM) for outcomes—from 1 to 3 years following the index venous thromboembolism (VTE) in patients under anticoagulation, Table S6. Association between thrombin generation parameters and venous thromboembolism (VTE) recurrence, major bleeding, and overall mortality—from 1 to 3 years following the index VTE in patients under anticoagulation, Figure S1. Thrombin generation parameters in patients under anticoagulation and not under anticoagulation 12 months after the index venous thromboembolism using 1 pM tissue factor without thrombomodulin (TM), Figure S2. Thrombin generation in patients under anticoagulation and not under anticoagulation 12 months after the index venous thromboembolism using 13.6 pM tissue factor without activated protein C (APC), Figure S3. Thrombin generation parameters in patients under anticoagulation and not under anticoagulation after index venous thromboembolism (VTE) using 1 pM tissue factor without thrombomodulin, Figure S4. Thrombin generation parameters in patients under anticoagulation and not under anticoagulation after index venous thromboembolism (VTE) using 13.6 pM tissue factor without activated protein C, Figure S5. Peak and endogenous thrombin potential (ETP) in patients under anticoagulation and not under anticoagulation after index venous thromboembolism (VTE) using 13.6 pM tissue factor with activated protein C (APC), Figure S6. Peak and endogenous thrombin potential (ETP) in patients under anticoagulation and not under anticoagulation after index venous thromboembolism using 1 pM tissue factor with thrombomodulin (TM), Figure S7. Thrombin generation parameters in patients under anticoagulation and not under anticoagulation after index venous thromboembolism using 13.6 pM tissue factor without activated protein C (APC), Figure S8. Thrombin generation parameters in patients under anticoagulation and not under anticoagulation after index venous thromboembolism using 13.6 pM tissue factor with activated protein C (APC), Figure S9. Thrombin generation parameters in patients under anticoagulation and not under anticoagulation after index venous thromboembolism using 1 pM tissue factor without thrombomodulin (TM), Figure S10. Peak and ETP in patients under anticoagulation and not under anticoagulation after index venous thromboembolism using 1 pM tissue factor, Figure S11. Thrombin generation parameters in patients under anticoagulation and not under anticoagulation after index venous thromboembolism using 13.6 pM tissue factor without activated protein C, Figure S12. Peak and endogenous thrombin potential (ETP) in patients under anticoagulation and not under anticoagulation after index venous thromboembolism using 13.6 pM tissue factor with activated protein C (APC).
