# Supplementary material for: Thrombin Generation Is Associated with Venous Thromboembolism Recurrence, but Not with Major Bleeding and Death in the Elderly: A Prospective Multicenter Cohort Study
Source: J Clin Med. 2023 Sep 19;12(18):6050. doi: 10.3390/jcm12186050 (PMC10531633; doi:10.3390/jcm12186050)
Supplement: Supplementary file 1 [file jcm-12-06050-s001.zip › jcm-2538048-supplementary.pdf]

SUPPLEMENTARY MATERIALS

TABLE

TABLE S1. Patient characteristics by thrombin generation results one year after index venous thromboembolism (VTE)

Characteristic*	All n (%) or median (IQ-range)	With thrombin generation result n (%) or median (IQ-range)	Without thrombin generation result n (%) or median (IQ-range)	*P*-value
**Total number of patients**	995	551	444	
**Patient age (years)**	75.0 (69.0; 81.0)	74.0 (69.0; 79.0)	76.0 (71.0; 83.0)	<0.001
**Female sex**	466 (47%)	232 (42%)	234 (53%)	0.001
**Index VTE event**				0.868
**PE only**	553 (56%)	309 (56%)	244 (55%)	
**DVT only**	307 (31%)	170 (31%)	137 (31%)	
**PE and DVT**	135 (14%)	72 (13%)	63 (14%)	
**Index DVT type***				0.018
**proximal DVT only**	197 (20%)	99 (18%)	98 (22%)	
**distal DVT only**	109 (11%)	56 (10%)	53 (12%)	
**proximal and distal DVT**	134 (13%)	87 (16%)	47 (11%)	
**Type of index VTE**				<0.001
**cancer-related VTE**	179 (18%)	60 (11%)	119 (27%)	
**provoked index VTE**	215 (22%)	113 (21%)	102 (23%)	
**unprovoked index VTE**	599 (60%)	378 (69%)	221 (50%)	
**Current oestrogen therapy during the last 3 months**	32 (3%)	18 (3%)	14 (3%)	1.000
**Immobilization during the last 3 months**	219 (22%)	95 (17%)	124 (28%)	<0.001
**Major surgery during the last 3 months**	150 (15%)	80 (15%)	70 (16%)	0.593
**Prior VTE**	283 (28%)	168 (30%)	115 (26%)	0.137
**PTS***	503 (51%)	291 (53%)	212 (48%)	0.173
**History of major bleeding**	101 (10%)	43 (8%)	58 (13%)	0.008
**Chronic liver disease**	14 (1%)	8 (1%)	6 (1%)	1.000
**Chronic renal disease**	185 (19%)	98 (18%)	87 (20%)	0.461
**Chronic or acute heart failure**	115 (12%)	65 (12%)	50 (11%)	0.842
**Cerebrovascular disease (stroke, TIA)**	92 (9%)	43 (8%)	49 (11%)	0.079
**Diabetes mellitus**	155 (16%)	87 (16%)	68 (15%)	0.930
**BMI >30 kg m^{-2}***	238 (24%)	136 (25%)	102 (23%)	0.653
**High risk of fall**	457 (46%)	234 (42%)	223 (50%)	0.010
**Acute rheumatic disease during the last 3 months**	32 (3%)	17 (3%)	15 (3%)	0.857
**Inflammatory bowel disease**	32 (3%)	17 (3%)	15 (3%)	0.857

**Severe infection or sepsis during the last 3 months**	83 (8%)	44 (8%)	39 (9%)	0.646
**Anemia***	389 (39%)	181 (33%)	208 (47%)	<0.001
**Platelet count <150 G L^{-1}***	140 (14%)	79 (14%)	61 (14%)	0.713
**Concomitant antiplatelet therapy**	322 (32%)	174 (32%)	148 (33%)	0.540
**Concomitant antiplatelet/NSAID therapy**	381 (38%)	204 (37%)	177 (40%)	0.358
**Arterial hypertension**	640 (64%)	356 (65%)	284 (64%)	0.947
**Heart rate ≥110 beats min^{-1}***	88 (9%)	46 (8%)	42 (9%)	0.577
**Systolic BP <100 mmHg***	35 (4%)	11 (2%)	24 (5%)	0.005
**Respiratory rate ≥30 min^{-1}***	33 (3%)	15 (3%)	18 (4%)	0.380
**Temperature <36C°***	70 (7%)	35 (6%)	35 (8%)	0.320
**Arterial oxygen saturation <90%***	107 (11%)	51 (9%)	56 (13%)	0.075
**D-dimer at the time of the index VTE***	2507.0 (1579.3; 3811.3)	2456.0 (1598.0; 3746.5)	2615.0 (1570.0; 3967.0)	0.399
**Factor VIII >164%***	573 (58%)	334 (61%)	239 (54%)	0.715
**Factor IX >134% (females) >138% (males***	157 (16%)	87 (16%)	70 (16%)	0.371
**Factor XI >139% (females) >138% (males)***	96 (10%)	53 (10%)	43 (10%)	0.510
**Fibrinogen >4.2 g L^{-1}***	511 (51%)	298 (54%)	213 (48%)	0.726
**Factor V Leiden mutation, present***	82 (8%)	53 (10%)	29 (7%)	0.290
**Factor II G20210A mutation, present***	49 (5%)	31 (6%)	18 (4%)	0.553
**Overall anticoagulation duration (days)**	413 (178; 896)	668.0 (213.0; 979.0)	246.0 (95.5; 639.5)	<0.001
**Anticoagulation duration until one year after the index VTE (days)**	324 (171; 368)	353.0 (194.0; 365.0)	232.5 (95.5; 407.5)	0.003
**Anticoagulation duration from one year after the index VTE (days)**	276 (0; 688)	344.0 (0.0; 693.0)	119.0 (0.0; 678.0)	0.059

BP, blood pressure; BMI, body mass index; DVT, deep vein thrombosis; IQR, interquartile range; NSAID, non-steroidal anti-inflammatory drug; PE, pulmonary embolism; PTS, post-thrombotic syndrome; TIA, transient ischemic attack. *Values were missing for index DVT type (56%), presence of PTS (3%), BMI > 30 kg m^{-2} (1%), anemia (7%), platelet count of <150 G L^{-1} (7%), heart rate of ≥ 110 beats min^{-1} (2%), systolic BP of < 100 mmHg (2%), respiratory rate of ≥ 30 min^{-1} (21%), temperature of < 36°C (8%), arterial oxygen saturation of < 90% (23%), D-dimer at the time of the index VTE (15%), factor VIII of > 164% (13%), factor IX of > 134% (females) and > 138% (males) (13%), factor XI of > 139% (women) and > 138% (males) (13%), fibrinogen of > 4.2 g/L (13%), factor V Leiden mutation present (11%), factor II G20210A mutation present (11%), anticoagulation duration from 12 months after the index VTE (34%).

TABLE S2. Characteristics of patients with results of thrombin generation with/without thrombomodulin one year after index venous thromboembolism (VTE)

Characteristic*	All patients n (%) or median (IQ-range)	Not under anticoagulation one year after index VTE n (%) or median (IQ-range)	Under anticoagulation one year after index VTE n (%) or median (IQ-range)	*P*-value
**Total number of patients**	535	222	313	
**Patient age (years)**	74.0 (69.o; 79.0)	74.0 (68.0; 78.0)	74.0 (69.0; 80.0)	0.400
**Female sex**	226 (42%)	103 (46%9	133 (39%)	0.110
**Index VTE event**				<0.001
**PE only**	298 (56%)	106 (48%)	192 (61%)	
**DVT only**	166 (31%)	95 (43%)	71 (23%)	
**PE and DVT**	71 (13%)	21 (9%)	50 (16%)	
**Index DVT type***	96 (18%)	44 (20%)	51 (17%)	<0.001
**proximal DVT only**	96 (18%)	44 (20%)	52 (17%)	
**distal DVT only**	56 (10%)	40 (18%)	16 (5%)	
**proximal and distal DVT**	85 (16%)	32 (14%)	53 (17%)	
**Type of index VTE**				<0.001
**cancer-related VTE**	59 (11%)	27 (12%)	32 (10%)	
**provoked index VTE**	109 (20%)	68 (31%)	41 (13%)	
**unprovoked index VTE**	367 (69%)	127 (57%)	240 (77%)	
**Current oestrogen therapy during the last 3 months**	17 (3%)	9 (4%)	8 (3%)	0.332
**Immobilization during the last 3 months**	94 (18%)	59 (27%)	35 (11%)	<0.001
**Major surgery during the last 3 months**	78 (15%)	52 (23%)	26 (8%)	<0.001
**Prior VTE**	160 (30%)	28 (13%)	132 (42%)	<0.001
**PTS***	286 (53%)	120 (54%)	166 (53%)	0.721
**History of major bleeding**	43 (8%)	18 (8%)	25 (8%)	1.000
**Chronic liver disease**	8 (1%)	4 (2%)	4 (1%)	0.724
**Chronic renal disease**	94 (18%)	33 (15%)	61 (19%)	0.205
**Chronic or acute heart failure**	60 (11%)	16 (7%)	44 (14%)	0.017
**Cerebrovascular disease (stroke, TIA)**	41 (8%)	13 (6%)	28 (9%)	0.248
**Diabetes mellitus**	85 (16%)	36 (16%)	49 (16%)	0.905
**BMI >30 kg m^{-2}***	131 (24%)	52 (23%)	79 (25%)	0.683
**High risk of fall**	225 (42%)	90 (41%)	135 (43%)	0.594
**Acute rheumatic disease during the last 3 months**	17 (3%)	9 (4%)	8 (3%)	0.332
**Inflammatory bowel disease**	17 (3%)	7 (3%)	10 (3%)	1.000
**Severe infection or sepsis during the last 3 months**	42 (8%)	16 (7%)	26 (8%)	0.745
**Anemia***	178 (33%)	83 (37%)	95 (30%)	0.035
**Platelet count <150 G L^{-1}***	78 (15%)	29 (13%)	49 (16%)	0.615

**Concomitant antiplatelet therapy**	167 (31%)	63 (28%)	104 (33%)	0.256
**Concomitant antiplatelet/NSAID therapy**	197 (37%)	74 (33%)	123 (39%)	0.173
**Arterial hypertension**	344 (64%)	140 (63%)	204 (65%)	0.647
**Heart rate ≥110 beats min^{-1}***	44 (8%)	14 (6%)	30 (10%)	0.205
**Systolic BP <100 mmHg***	11 (2%)	3 (1%)	8 (3%)	0.538
**Respiratory rate ≥30 min^{-1}***	14 (3%)	4 (2%)	10 (3%)	0.411
**Temperature <36C°***	34 (6%)	18 (8%)	16 (5%)	0.106
**Arterial oxygen saturation <90%***	50 (9%)	15 (7%)	35 (11%)	0.278
**D-dimer at the time of the index VTE***	2486.0 (1598.0; 3757.0)	2404.0 (1677.0; 3765.0)	2514.5 (1554.0; 3744.0)	0.980
**Factor VIII >164%***	326 (61%)	126 (57%)	200 (64%)	0.123
**Factor IX >134% (females) >138% (males***	82 (15%)	42 (19%)	40 (13%)	0.050
**Factor XI >139% (females) >138% (males)***	49 (9%)	21 (9%)	28 (9%)	0.879
**Fibrinogen >4.2 g L^{-1}***	288 (54%)	113 (51%)	175 (56%)	0.355
**Factor V Leiden mutation, present***	49 (9%)	19 (9%)	30 (10%)	<0.001
**Factor II G20210A mutation, present***	30 (6%)	9 (4%)	21 (7%)	<0.001
**Overall anticoagulation duration (days)**	658.0 (210.0; 977.0)	191.5 (144.0; 283.0)	900.0 (710.0; 1225.5)	<0.001
**Anticoagulation duration until one year after the index VTE (days)**	353.0 (194.0; 365.0)	185.0 (119.5; 210.8)	364.0 (357.0; 371.0)	<0.001
**Anticoagulation duration from one year after the index VTE (days)**	344.0 (0.0; 691.0)	0.0 (0.0; 0.0)	535.0 (353.0; 871.0)	<0.001

BP, blood pressure; BMI, body mass index; DVT, deep vein thrombosis; IQR, interquartile range; NSAID, non-steroidal anti-inflammatory drug*;* PE, pulmonary embolism; PTS, post-thrombotic syndrome; TIA, transient ischemic attack. *Values were missing for index DVT type (56%), presence of PTS (2%), anemia (8%), platelet count of <150 G L^{-1} (8%), heart rate of ≥ 110 beats min^{-1} (3%), systolic BP of < 100 mmHg (2%), respiratory rate of ≥ 30 min^{-1} (25%), temperature of < 36°C (7%), arterial oxygen saturation of < 90% (24%), D-dimer at the time of the index VTE (8%), factor VIII of > 164% (7%), factor IX of > 134% (females) and > 138% (males) (7%), factor XI of > 139% (women) and > 138% (males) (7%), fibrinogen of > 4.2 g/L (7%), factor V Leiden mutation present (5%), factor II G20210A mutation present (5%).

TABLE S3. Characteristics of patients with results of normalized assays with/without thrombomodulin one year after index venous thromboembolism (VTE)

Characteristic*	All patients n (%) or median (IQ-range)	Not under anticoagulation one year after index VTE n (%) or median (IQ-range)	Under anticoagulation one year after index VTE n (%) or median (IQ-range)	*P*-value
**Total number of patients**	320	122	198	
**Patient age (years)**	73.0 (68.0; 78.8)	73.0 (68.0; 79.0)	73.0 (68.0; 78.0)	0.972
**Female sex**	124 (39%)	55 (45%)	69 (35%)	0.077
**Index VTE event**				<0.001
**PE only**	182 (57%)	57 (47%)	125 (63%)	
**DVT only**	96 (30%)	55 (45%)	41 (21%)	
**PE and DVT**	42 (13%)	10 (8%)	32 (16%)	
**Index DVT type***				0.001
**proximal DVT only**	52 (16%)	20 (16%)	32 (16%)	
**distal DVT only**	37 (12%)	27 (22%)	10 (5%)	
**proximal and distal DVT**	49 (15%)	18 (15%)	31 (16%)	
**Type of index VTE**				<0.001
**cancer-related VTE**	42 (13%)	17 (14%)	25 (13%)	
**provoked index VTE**	66 (21%)	42 (34%)	24 (12%)	
**unprovoked index VTE**	212 (66%)	63 (52%)	149 (75%)	
**Current oestrogen therapy during the last 3 months**	9 (3%)	5 (4%)	4 (2%)	0.310
**Immobilization during the last 3 months**	59 (18%)	37 (30%)	22 (11%)	<0.001
**Major surgery during the last 3 months**	49 (15%)	33 (27%)	16 (8%)	<0.001
**Prior VTE**	106 (33%)	19 (16%)	87 (44%)	<0.001
**PTS***	163 (51%)	60 (49%)	103 (52%)	0.907
**History of major bleeding**	27 (8%)	12 (10%)	15 (8%)	0.537
**Chronic liver disease**	6 (2%)	2 (2%)	4 (2%)	1.000
**Chronic renal disease**	58 (18%)	22 (18%)	36 (18%)	1.000
**Chronic or acute heart failure**	28 (9%)	6 (5%)	22 (11%)	0.067
**Cerebrovascular disease (stroke, TIA)**	25 (8%)	8 (7%)	17 (9%)	0.669
**Diabetes mellitus**	46 (14%)	17 (14%)	29 (15%)	1.000
**BMI >30 kg m^{-2}***	77 (24%)	26 (21%)	51 (26%)	0.420
**High risk of fall**	124 (39%)	45 (37%)	79 (40%)	0.637
**Acute rheumatic disease during the last 3 months**	11 (3%)	5 (4%)	6 (3%)	0.754
**Inflammatory bowel disease**	9 (3%)	5 (4%)	4 (2%)	0.310
**Severe infection or sepsis during the last 3 months**	29 (9%)	10 (8%)	19 (10%)	0.841
**Anemia***	109 (34%)	48 (39%)	61 (31%)	0.045
**Platelet count <150 G L^{-1}***	52 (16%)	15 (12%)	37 (19%)	0.208
**Concomitant antiplatelet therapy**	99 (31%)	36 (30%)	63 (32%)	0.710

**Concomitant antiplatelet/NSAID therapy**	113 (35%)	38 (31%)	75 (38%)	0.231
**Arterial hypertension**	194 (61%)	76 (62%)	118 (60%)	0.640
**Heart rate ≥110 beats min^{-1}***	26 (8%)	7 (6%)	19 (10%)	0.294
**Systolic BP <100 mmHg***	5 (2%)	1 (1%)	4 (2%)	0.654
**Respiratory rate ≥30 min^{-1}***	9 (3%)	3 (2%)	6 (3%)	1.000
**Temperature <36C°***	21 (7%)	9 (7%)	12 (6%)	0.490
**Arterial oxygen saturation <90%***	32 (10%)	9 (7%)	23 (12%)	0.433
**D-dimer at the time of the index VTE***	2394.0 (1519.3; 3766.3)	2386.5 (1508.3; 3875.8)	2401.0 (1518.3; 3696.8)	0.938
**Factor VIII >164%***	204 (64%)	73 (60%)	131 (66%)	0.428
**Factor IX >134% (females) >138% (males***	45 (14%)	19 (16%)	26 (13%)	0.505
**Factor XI >139% (females) >138% (males)***	30 (9%)	13 (11%)	17 (9%)	0.551
**Fibrinogen >4.2 g L^{-1}***	171 (53%)	59 (48%)	112 (57%)	0.269
**Factor V Leiden mutation, present***	27 (8%)	7 (6%)	20 (10%)	0.215
**Factor II G20210A mutation, present***	16 (5%)	3 (2%)	13 (7%)	0.120
**Overall anticoagulation duration (days)**	606.5 (211.0; 896.5)	191.0 (120.8; 253.0)	731.5 (665.5; 952.5)	<0.001
**Anticoagulation duration until one year after the index VTE (days)**	355.5 (199.3; 366.0)	183.5 (116.0; 210.0)	364.0 (357.0; 371.3)	<0.001
**Anticoagulation duration from one year after the index VTE (days)**	262.0 (0.0; 521.0)	0.0 (0.0; 0.0)	376.5 (337.8; 592.5)	<0.001

BP, blood pressure; BMI, body mass index; DVT, deep vein thrombosis; IQR, interquartile range; NSAID, non-steroidal anti-inflammatory drug; PE, pulmonary embolism; PTS, post-thrombotic syndrome; TIA, transient ischemic attack. *Values were missing for index DVT type (57%), presence of PTS (2%), anemia (8%), platelet count of <150 G L^{-1} (8%), heart rate of ≥ 110 beats min^{-1} (3%), systolic BP of < 100 mmHg (2%), respiratory rate of ≥ 30 min^{-1} (26%), temperature of < 36°C (7%), arterial oxygen saturation of < 90% (22%), D-dimer at the time of the index VTE (9%), factor VIII of > 164% (9%), factor IX of > 134% (females) and > 138% (males) (9%), factor XI of > 139% (women) and > 138% (males) (9%), fibrinogen of > 4.2 g/L (9%), factor V Leiden mutation present (6%), factor II G20210A mutation present (6%).

TABLE S4. Incidence rate of venous thromboembolism (VTE) recurrence, major bleeding, or overall mortality per 100 person-years - from 12 months until 36 months after index VTE in anticoagulated patients.

		No of patients	No of events/ person-years	Incidence rate (95%-CI)
**Peak ratio obtained in presence/absence of TM**				
VTE recurrence				
	All	307	10 / 455.2	2.2 (1.2 to 4.1)
	≤ median	154	9 / 229.6	3.9 (2.0 to 7.5)
	> median	153	1 / 225.6	0.4 (0.1 to 3.1)
Major bleeding				
	All	307	13 / 455.7	2.9 (1.7 to 4.9)
	≤ median	154	5 / 233.9	2.1 (0.9 to 5.1)
	> median	153	8 / 221.8	3.6 (1.8 to 7.2)
Overall mortality				
	All	307	21 / 461.5	4.6 (3.0 to 7.0)
	≤ median	154	10 / 235.5	4.2 (2.3 to 7.9)
	> median	153	11 / 226.0	4.9 (2.7 to 8.8)
**Normalized peak ratio in presence/absence of TM**				
VTE recurrence				
	All	198	3 / 259.1	1.2 (0.4 to 3.6)
	≤ median	99	2 / 127.8	1.6 (0.4 to 6.3)
	> median	99	1 / 131.3	0.8 (0.1 to 5.4)
Major bleeding				
	All	198	9 / 256.9	3.5 (1.8 to 6.7)
	≤ median	99	2 / 129.7	1.5 (0.4 to 6.2)
	> median	99	7 / 127.3	5.5 (2.6 to 11.5)
Overall mortality				
	All	198	11 / 260.9	4.2 (2.3 to 7.6)
	≤ median	99	4 / 129.7	3.1 (1.2 to 8.2)
	> median	99	7 / 131.3	5.3 (2.5 to 11.2)
**ETP ratio obtained in presence/absence of TM**				
VTE recurrence				

	All	307	10 / 455.2	2.2 (1.2 to 4.1)
	≤ median	154	7 / 231.2	3.0 (1.4 to 6.4)
	> median	153	3 / 224.0	1.3 (0.4 to 4.2)
Major bleeding				
	All	307	13 / 455.7	2.9 (1.7 to 4.9)
	≤ median	154	5 / 235.1	2.1 (0.9 to 5.1)
	> median	153	8 / 220.6	3.6 (1.8 to 7.3)
Overall mortality				
	All	307	21 / 461.5	4.6 (3.0 to 7.0)
	≤ median	154	11 / 236.7	4.6 (2.6 to 8.4)
	> median	153	10 / 224.8	4.4 (2.4 to 8.3)
**Normalized ETP ratio obtained in presence/absence of TM**				
VTE recurrence				
	All	198	3 / 259.1	1.2 (0.4 to 3.6)
	≤ median	99	1 / 126.6	0.8 (0.1 to 5.6)
	> median	99	2 / 132.5	1.5 (0.4 to 6.0)
Major bleeding				
	All	198	9 / 256.9	3.5 (1.8 to 6.7)
	≤ median	99	3 / 127.7	2.3 (0.8 to 7.3)
	> median	99	6 / 129.3	4.6 (2.1 to 10.3)
Overall mortality				
	All	198	11 / 260.9	4.2 (2.3 to 7.6)
	≤ median	99	5 / 128.0	3.9 (1.6 to 9.4)
	> median	99	6 / 132.9	4.5 (2.0 to 10.0)

All experiments have been conducted in presence of 1 pM tissue factor (TF)

TABLE S5. Discriminative power of thrombin generation parameters involving thrombomodulin (TM) for outcomes – from 1 year to 3 years following the index venous thromboembolisme (VTE) in patients under anticoagulation.

Thrombin generation parameters measured one year after the index VTE	No. of events/no. of patients	*C*-statistics (95% confidence interval)
**Peak ratio obtained in presence/absence of TM**		
VTE recurrence	10/307	0.74 (0.60 to 0.88)
Major bleeding	13/307	0.60 (0.45 to 0.74)
Overall mortality	21/307	0.56 (0.42 to 0.70)
**Normalized peak ratio obtained in presence/absence of TM**		
VTE recurrence	3/198	0.69 (0.37 to 1.02)
Major bleeding	9/198	0.60 (0.46 to 0.74)
Overall mortality	11/198	0.46 (0.24 to 0.68)
**ETP ratio obtained in presence/absence of TM**		
VTE recurrence	10/307	0.65 (0.45 to 0.84)
Major bleeding	13/307	0.56 (0.41 to 0.71)
Overall mortality	21/307	0.55 (0.39 to 0.70)
**Normalized ETP ratio obtained in presence/absence of TM**		
VTE recurrence	3/198	0.56 (0.11 to 1.01)
Major bleeding	9/198	0.59 (0.43 to 0.75)
Overall mortality	11/198	0.49 (0.26 to 0.71)

All experiments have been conducted in presence of 1 pM tissue factor

TABLE S6. Association between thrombin generation parameters and venous thromboembolism (VTE) recurrence, major bleeding and overall mortality – from 1 year to 3 years following the index VTE in patients under anticoagulation.

	n/N (%)	Crude subhazard ratio (95% confidence interval)	Adjusted subhazard ratio (95% confidence interval)
Peak ratio obtained in presence/absence of TM (TF 1 pM)			
VTE recurrence	10/307 (3.3)	0.11 (0.03 to 0.45)	0.12 (0.03 to 0.51)
Major bleeding	13/307 (4.2)	1.15 (0.87 to 1.54)	1.15 (0.87 to 1.54)
Overall mortality	21/307 (6.8)	0.34 (0.05 to 2.10)	0.34 (0.05 to 2.10)
Normalized peak ratio obtained in presence/absence of TM (TF 1 pM)			
VTE recurrence	3/198 (1.5)	0.37 (0.08 to 1.78)	0.38 (0.07 to 2.06)
Major bleeding	9/198 (4.5)	1.07 (0.90 to 1.26)	-
Overall mortality	11/198 (5.6)	0.91 (0.43 to 1.90)	-
ETP ratio obtained in presence/absence of TM (TF 1 pM)			
VTE recurrence	10/307 (3.3)	0.14 (0.02 to 1.01)	0.13 (0.02 to 0.90)
Major bleeding	13/307 (4.2)	1.66 (0.73 to 3.82)	1.72 (0.74 to 4.00)
Overall mortality	21/307 (6.8)	0.29 (0.03 to 2.39)	0.25 (0.03 to 2.08)
Normalized ETP ratio obtained in presence/absence of APC (TF 13.6 pM)			
VTE recurrence	11/290 (3.8)	1.09 (0.91 to 1.31)	1.13 (0.95 to 1.34)
Major bleeding	15/290 (5.2)	1.05 (0.89 to 1.25)	1.08 (0.90 to 1.29)
Overall mortality	22/290 (7.6)	0.79 (0.54 to 1.16)	0.92 (0.70 to 1.21)

Abbreviations: APC, activated protein C; ETP, endogenous thrombin potential; TM, thrombomodulin. Adjustments: VTE recurrence was adjusted for age, cancer, provoked VTE, prior VTE, overt pulmonary embolism, renal disease and periods of anticoagulation (oral or parenteral anticoagulation) as a time-varying covariable.[1-10] Major bleeding was adjusted for age, cancer, provoked VTE, prior VTE, overt pulmonary embolism, renal disease, history of major bleeding, anemia, antiplatelet therapy and periods of anticoagulation as time-varying covariate.[11-26] Mortality was adjusted for age, gender, cancer, provoked VTE, prior VTE, overt pulmonary embolism, renal disease, history of major bleeding, heart failure, chronic lung disease, high pulse, low blood pressure, low oxygen, and periods of anticoagulation as a time-varying covariate.[7, 27]

FIGURES

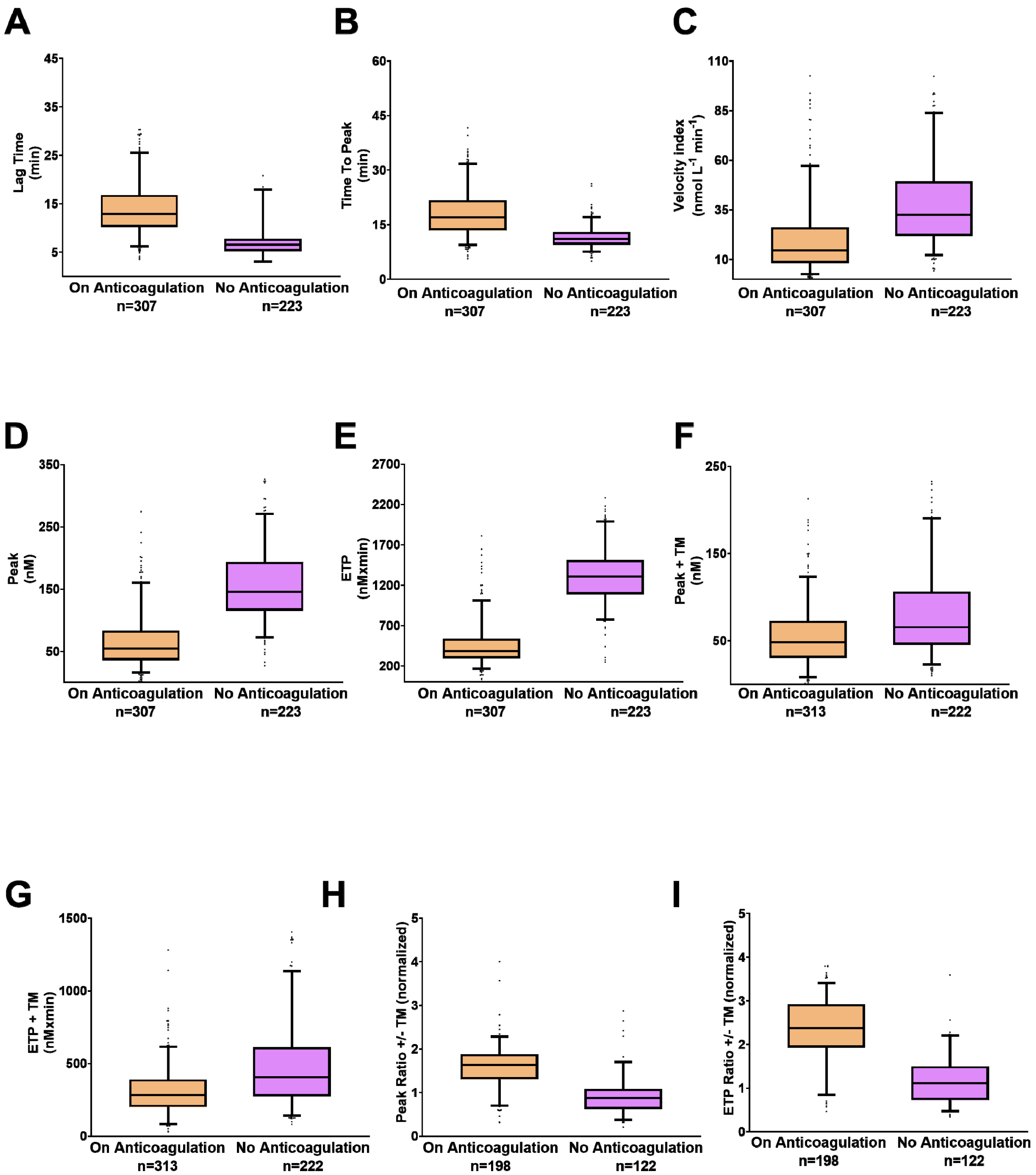

FIGURE S1. Thrombin generation parameters in patients under anticoagulation and not under anticoagulation 12 months after the index venous thromboembolism using 1pM tissue factor without thrombomodulin (TM) (A-E), with TM (F-G) and normalized ratio with and without TM for peak (H) and endogenous thrombin potential (ETP) (I). Box-plots of thrombin generation parameters are presented as median with interquartile range (5-95%).

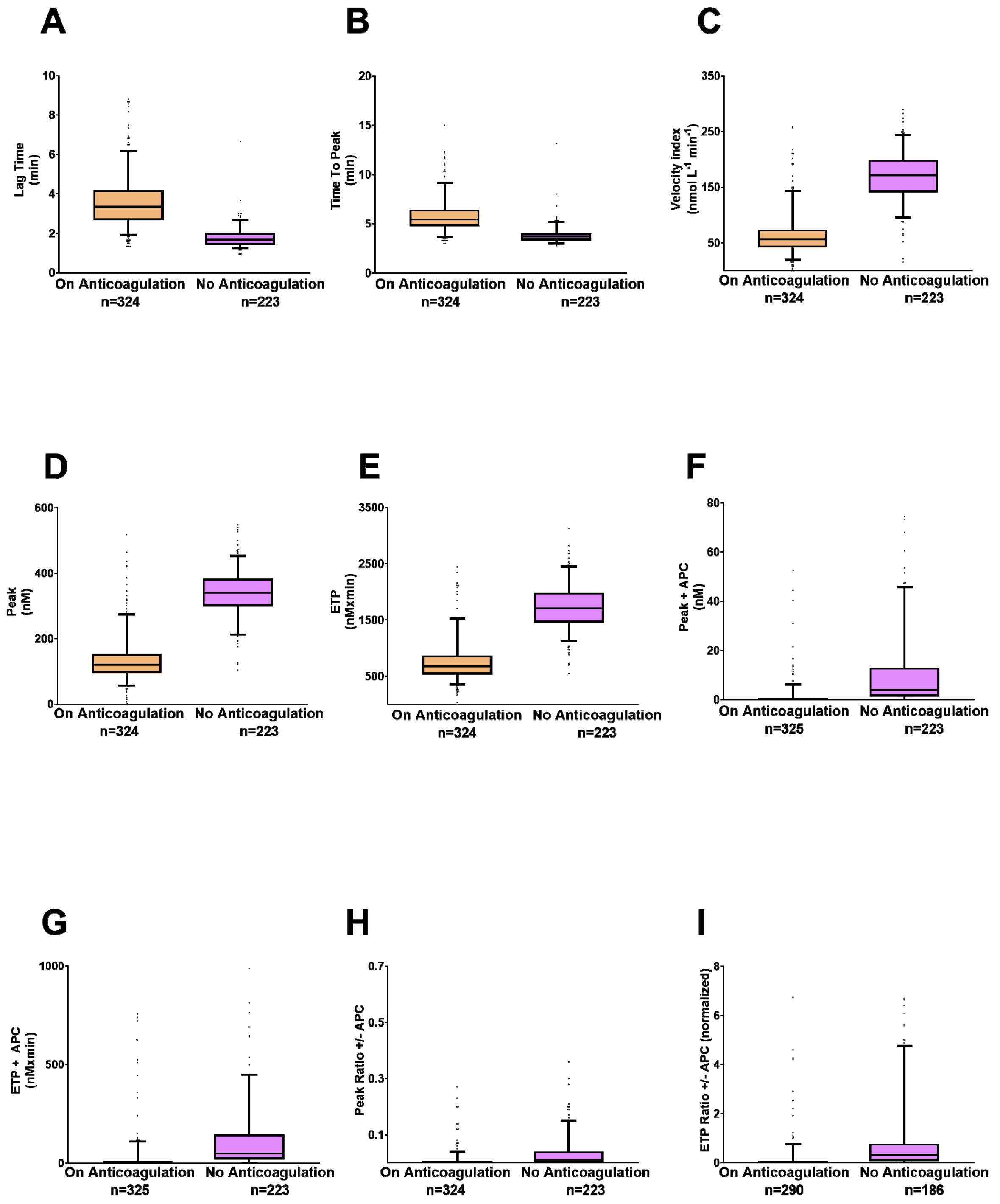

FIGURE S2. Thrombin generation in patients under anticoagulation and not under anticoagulation 12 months after the index venous thromboembolism using 13.6 pM tissue factor without activated protein C (APC) (A-E), with APC (F-G) and ratio with and without APC for peak (H) and normalized ratio with and without APC for endogenous thrombin potential (ETP) (I). Box-plots of thrombin generation parameters are presented as median with interquartile range (5-95%).

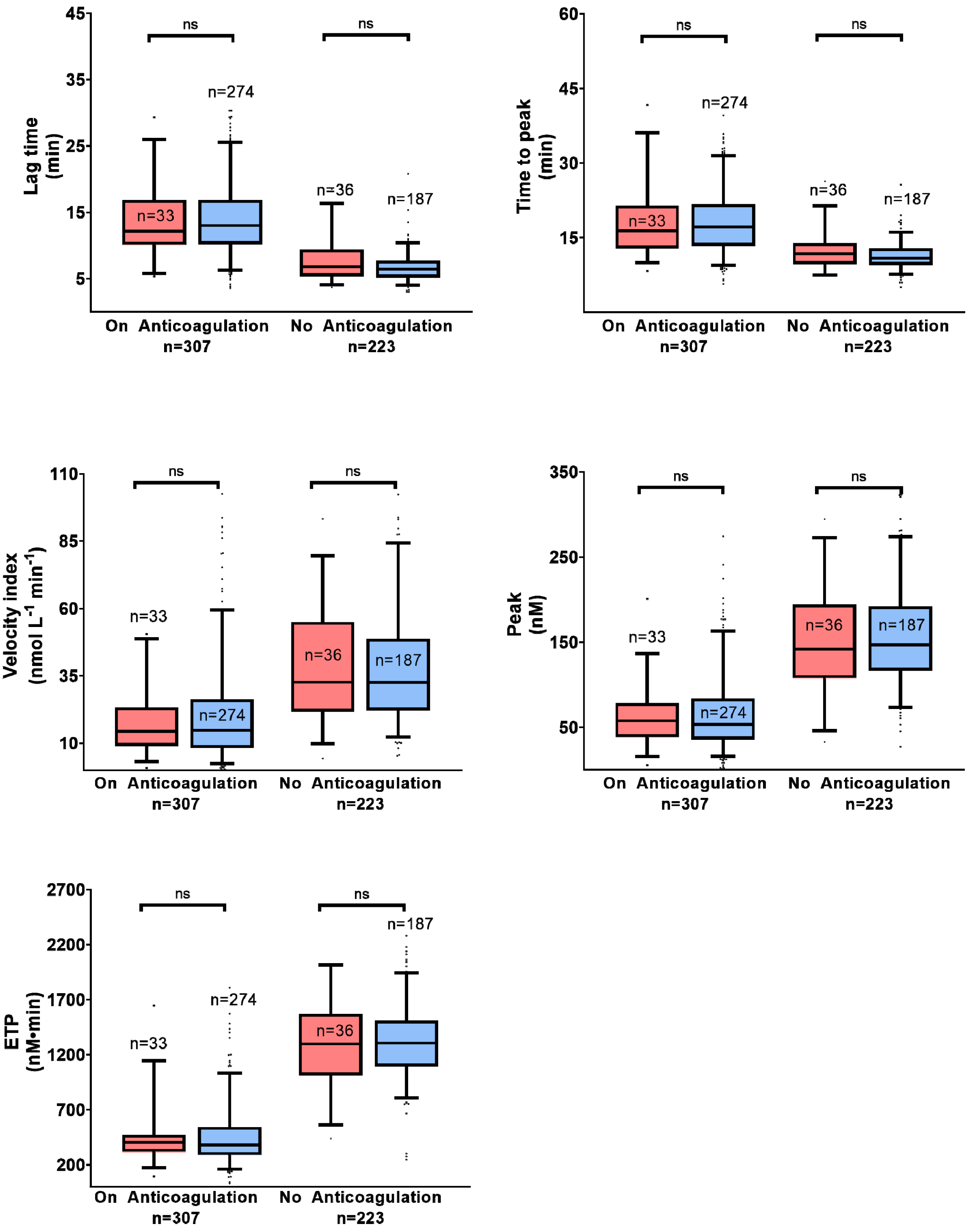

Figure S3. Thrombin generation parameters in patients under anticoagulation and not under anticoagulation after index venous thromboembolism (VTE) using 1 pM tissue factor without thrombomodulin. The red boxes indicate patients with VTE recurrence and the blue boxes, those without VTE recurrence up to 24 months following the index VTE. Box-plots of thrombin generation parameters are presented as median with interquartile range (5-95%). Groups were compared using Mann-Whitney U test. ETP, endogenous thrombin potential; ns, not significant

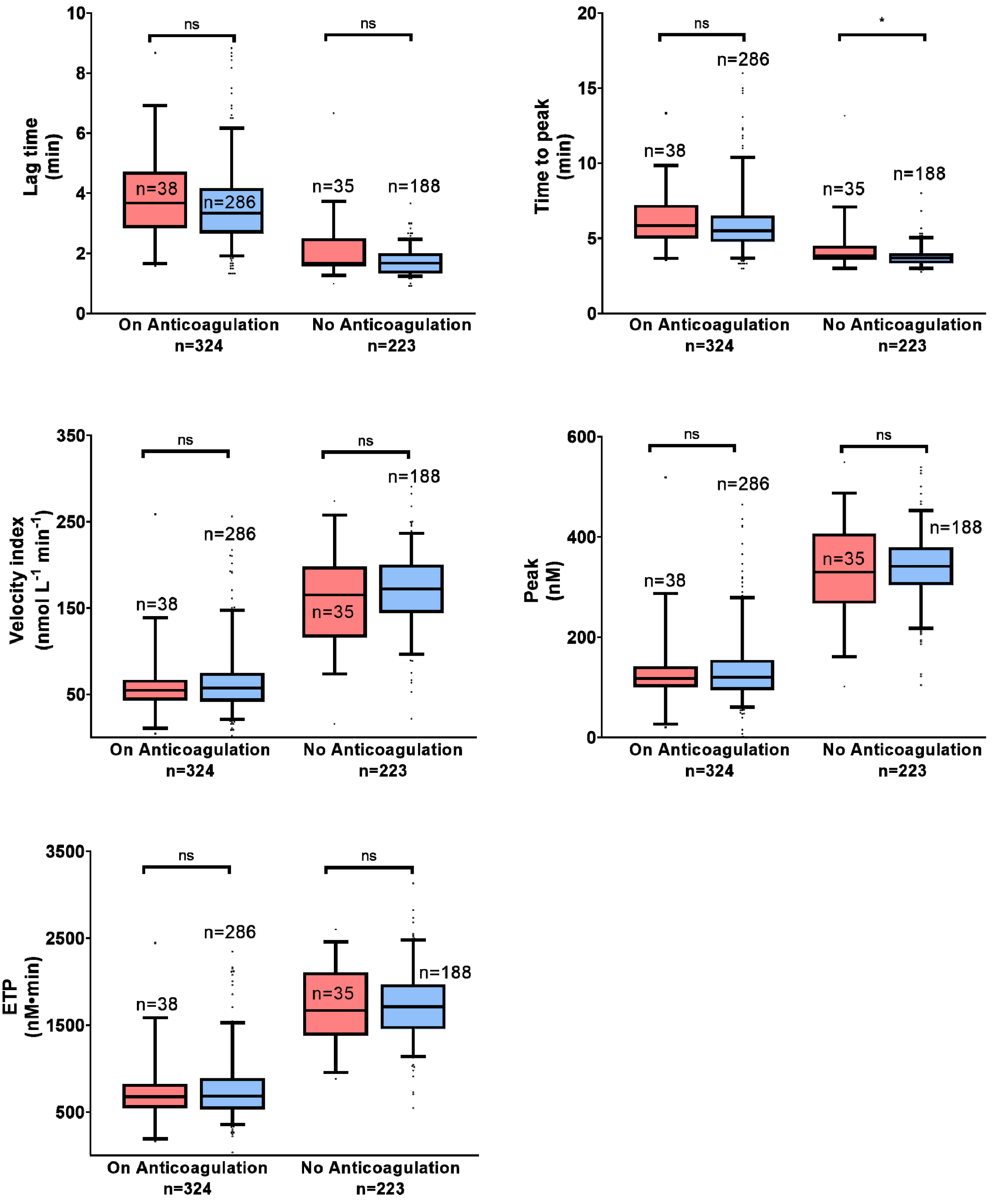

Figure S4. Thrombin generation parameters in patients under anticoagulation and not under anticoagulation after index venous thromboembolim (VTE) using 13.6 pM tissue factor without activated protein C. The red boxes indicate patients with VTE recurrence and the blue boxes, those without VTE recurrence up to 24 months following the index VTE. Box-plots of thrombin generation parameters are presented as median with interquartile range (5-95%). Groups were compared using the Mann-Whitney U test. ETP, endogenous thrombin potential; ns, not significant; *P<0.05

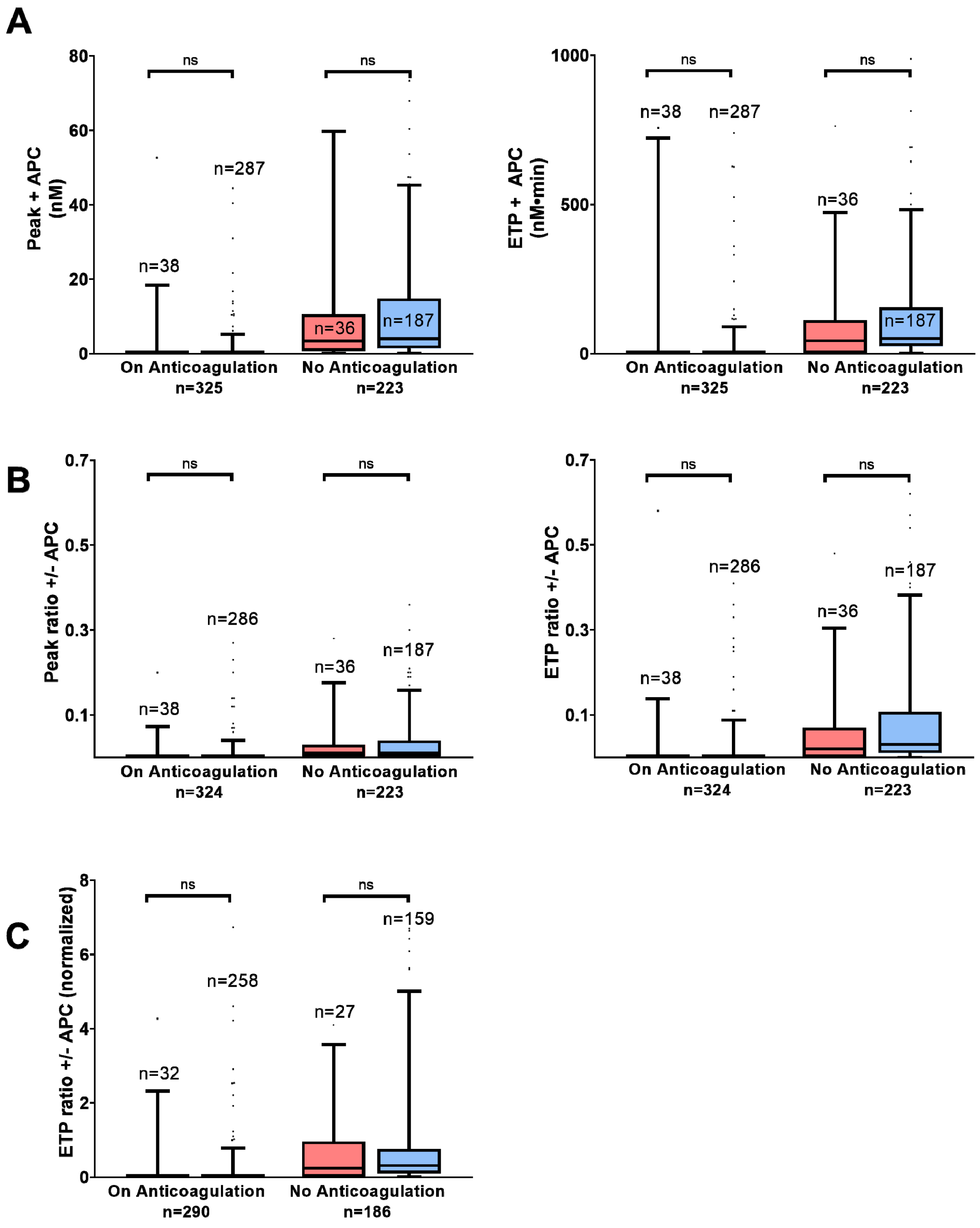

Figure S5. Peak and endogenous thrombin potential (ETP) in patients under anticoagulation and not under anticoagulation after index venous thromboembolim (VTE) using 13.6 pM tissue factor with activated protein C (APC) (A), peak and ETP ratio with and without APC (B), ETP with and without APC normalized with reference plasma (C). The red boxes indicate patients with VTE recurrence and the blue boxes, those without VTE recurrence up to 24 months following the index VTE. Box-plots of thrombin generation parameters presented as median with interquartile range (5-95%). Groups were compared using Mann-Whitney U test. ns, not significant

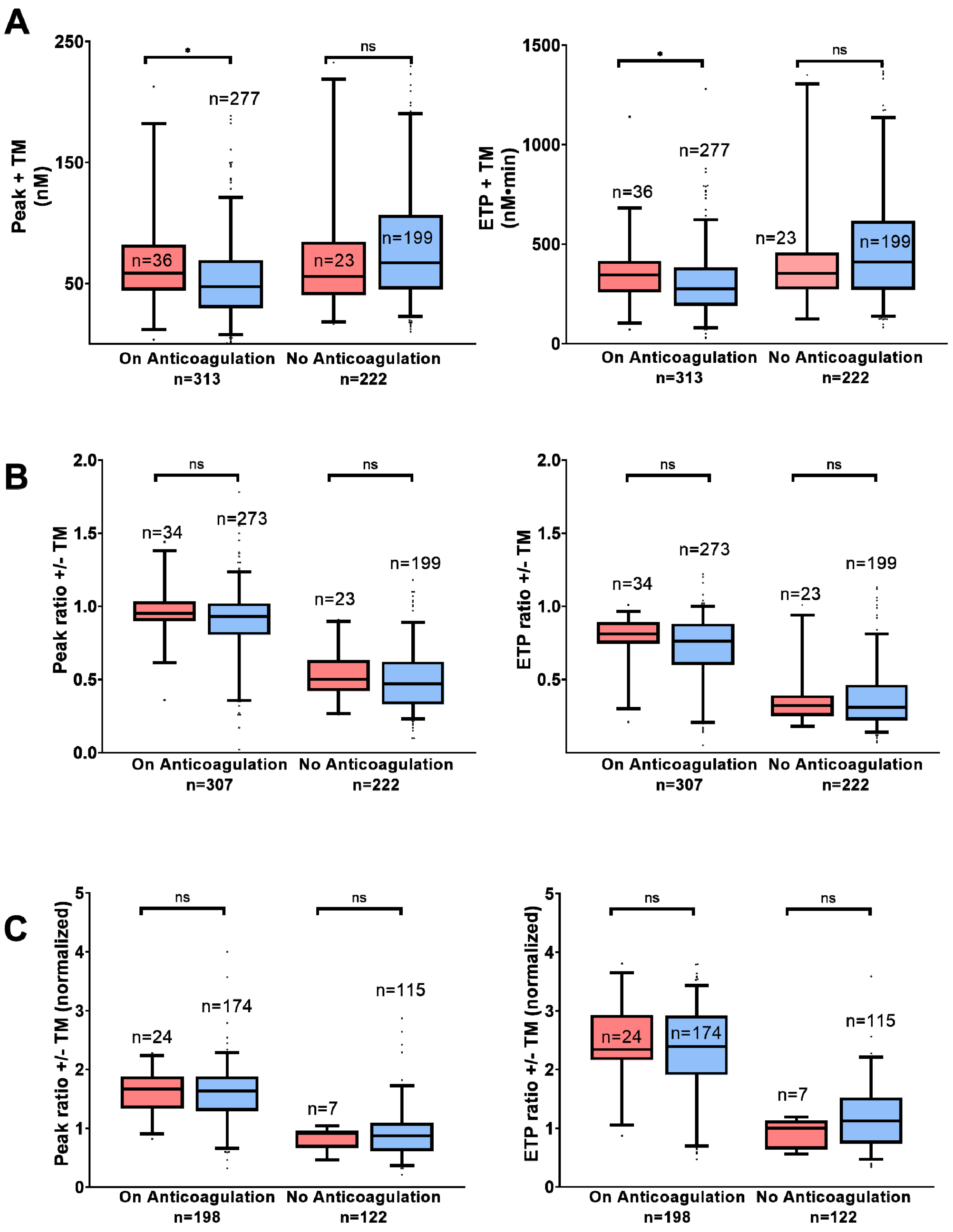

Figure S6. Peak and endogenous thrombin potential (ETP) in patients under anticoagulation and not under anticoagulation after index venous thromboembolism using 1 pM tissue factor with thrombomodulin (TM) (A); ratio (B) and normalized ratio (C) for peak and ETP with and without TM. The red boxes indicate patients who had a major bleeding event and the blue boxes, those without major bleeding up to 24 months following the index VTE. Box-plots of thrombin generation parameters are presented as median with interquartile range (5-95%) are indicated. Groups were compared using the Mann-Whitney U test. ns, not significant; *P<0.05

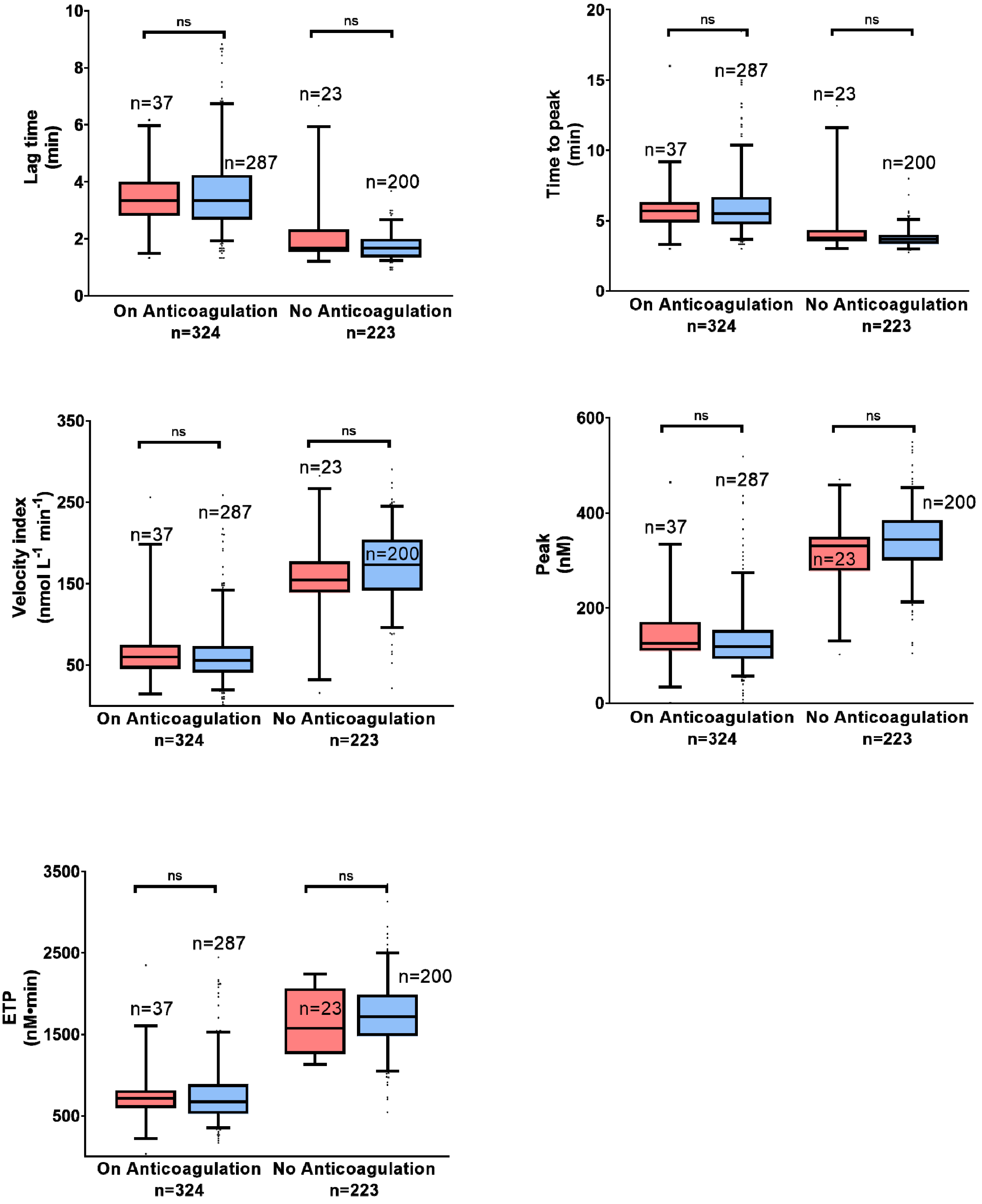

Figure S7. Thrombin generation parameters in patients under anticoagulation and not under anticoagulation after index venous thromboembolim using 13.6 pM tissue factor without activated protein C (APC). The red boxes indicate patients who had a major bleeding event and the blue boxes, those without major bleeding up to 24 months following the index VTE. Box-plots of thrombin generation parameters are presented as median with interquartile range (5-95%) are indicated. Groups were compared using the Mann-Whitney U test. ETP, endogenous thrombin potential, ns, not significant

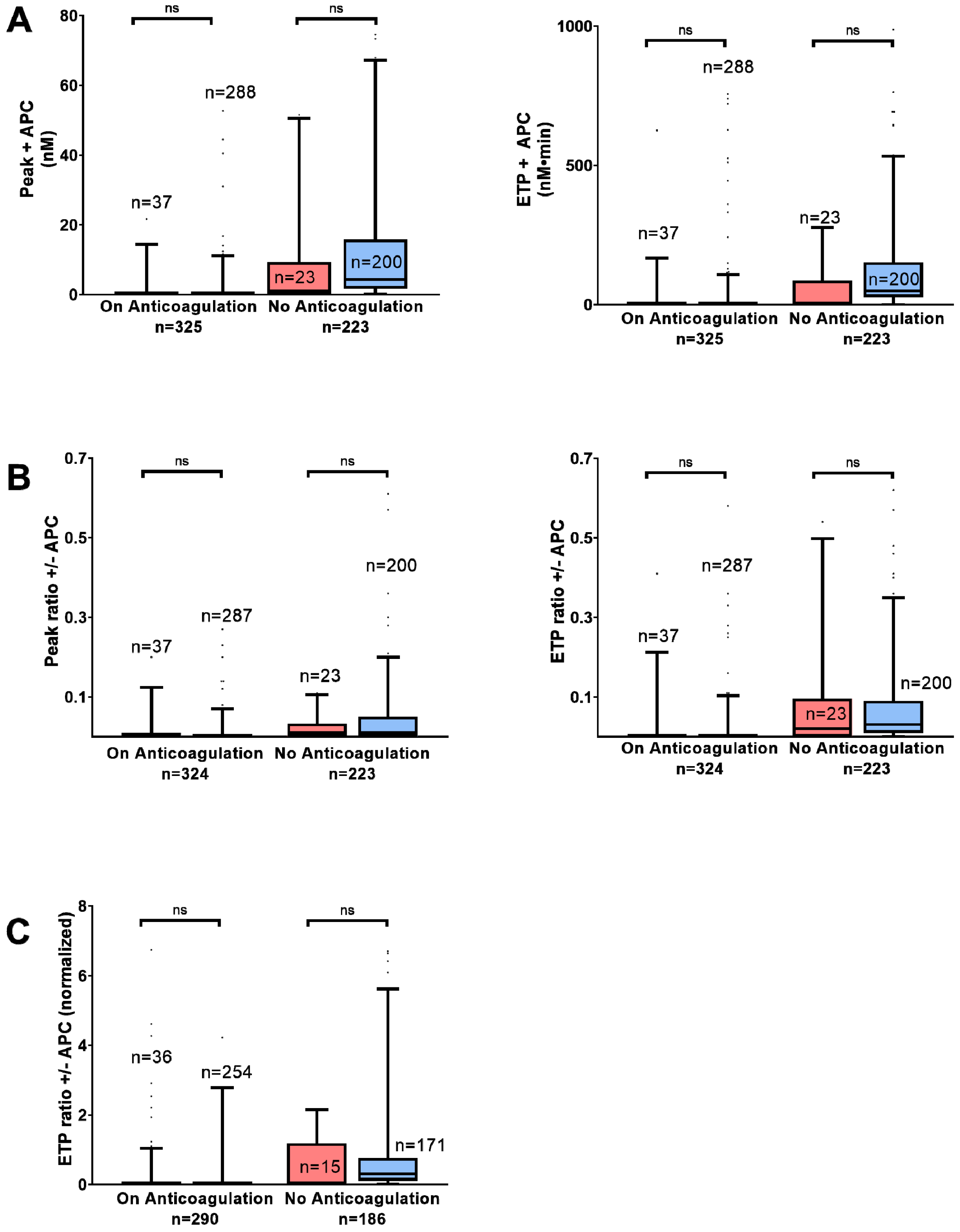

Figure S8. Thrombin generation parameters in patients under anticoagulation and not under anticoagulation after index venous thromboembolim using 13.6 pM tissue factor with activated protein C (APC) (A), calculated ratio for peak and endogenous thrombin potential (ETP) (B) and normalized ratio (C) for ETP with and without APC. The red boxes indicate patients who had a major bleeding event and the blue boxes, those without major bleeding up to 24 months following the index VTE. Box-plots of thrombin generation parameters are presented as median with interquartile range (5-95%) are indicated. Groups were compared using the Mann-Whitney U test. ns, not significant

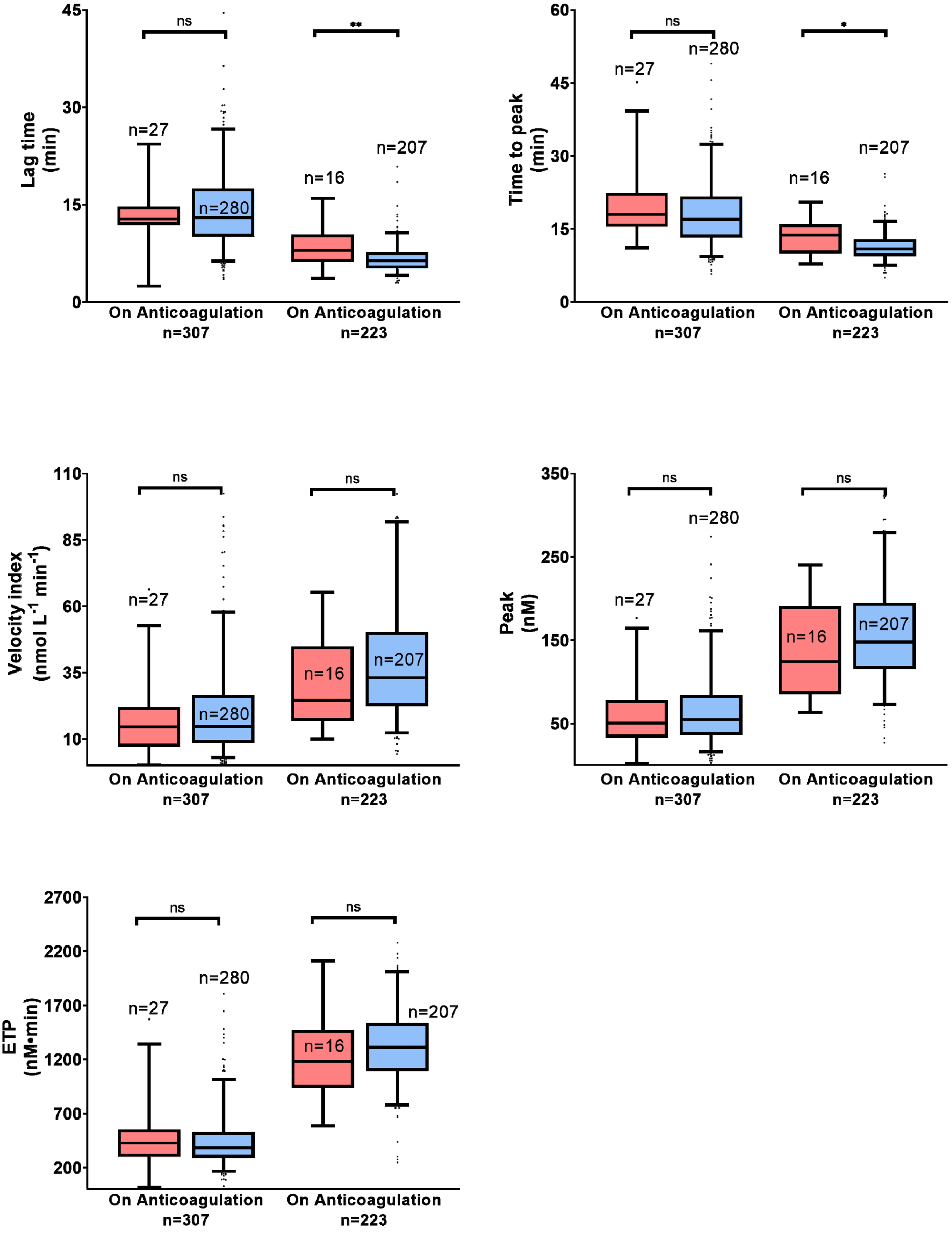

Figure S9. Thrombin generation parameters in patients under anticoagulation and not under anticoagulation after index venous thromboembolim using 1 pM tissue factor without thrombomodulin (TM). The red boxes indicate patients who died and the blue boxes, those who did not up to 24 months following the index VTE. Box-plots of thrombin generation parameters are presented as median with interquartile range (5-95%) are indicated. Groups were compared using the Mann-Whitney U test. ETP, endogenous thrombin potential; ns, not significant; **P<0.01, *P<0.05

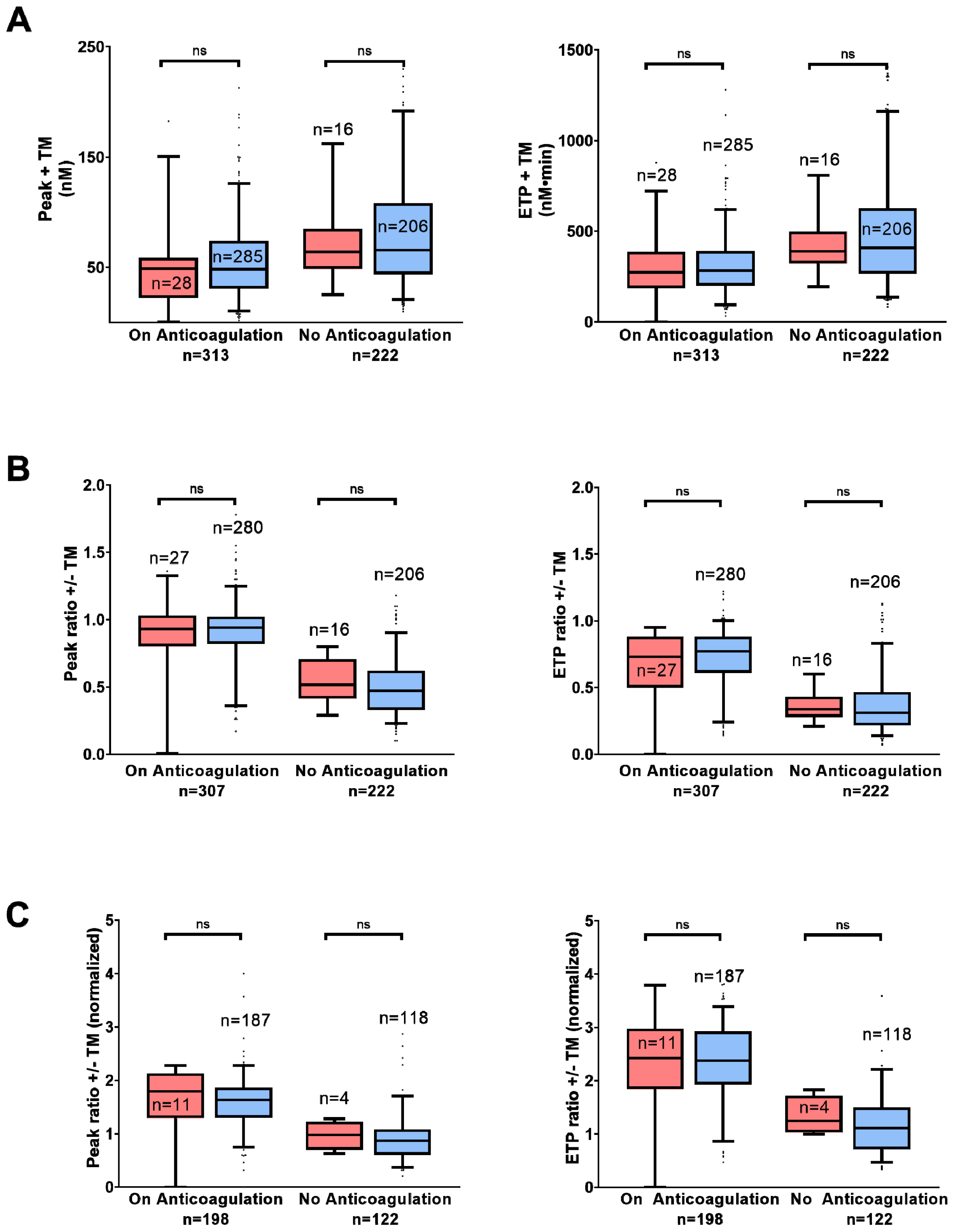

Figure S10. Peak and ETP in patients under anticoagulation and not under anticoagulation after index venous thromboembolim using 1 pM tissue factor with thrombomodulin (TM) (A), calculated ratio (B) and normalized ratio (C) for peak and endogenous thrombin potential (ETP) with and without TM. The red boxes indicate patients who died and the blue boxes, those who did not up to 24 months following the index VTE. Box-plots of thrombin generation parameters are presented as median with interquartile range (5-95%) are indicated. Groups were compared using the Mann-Whitney U test. ns, not significant

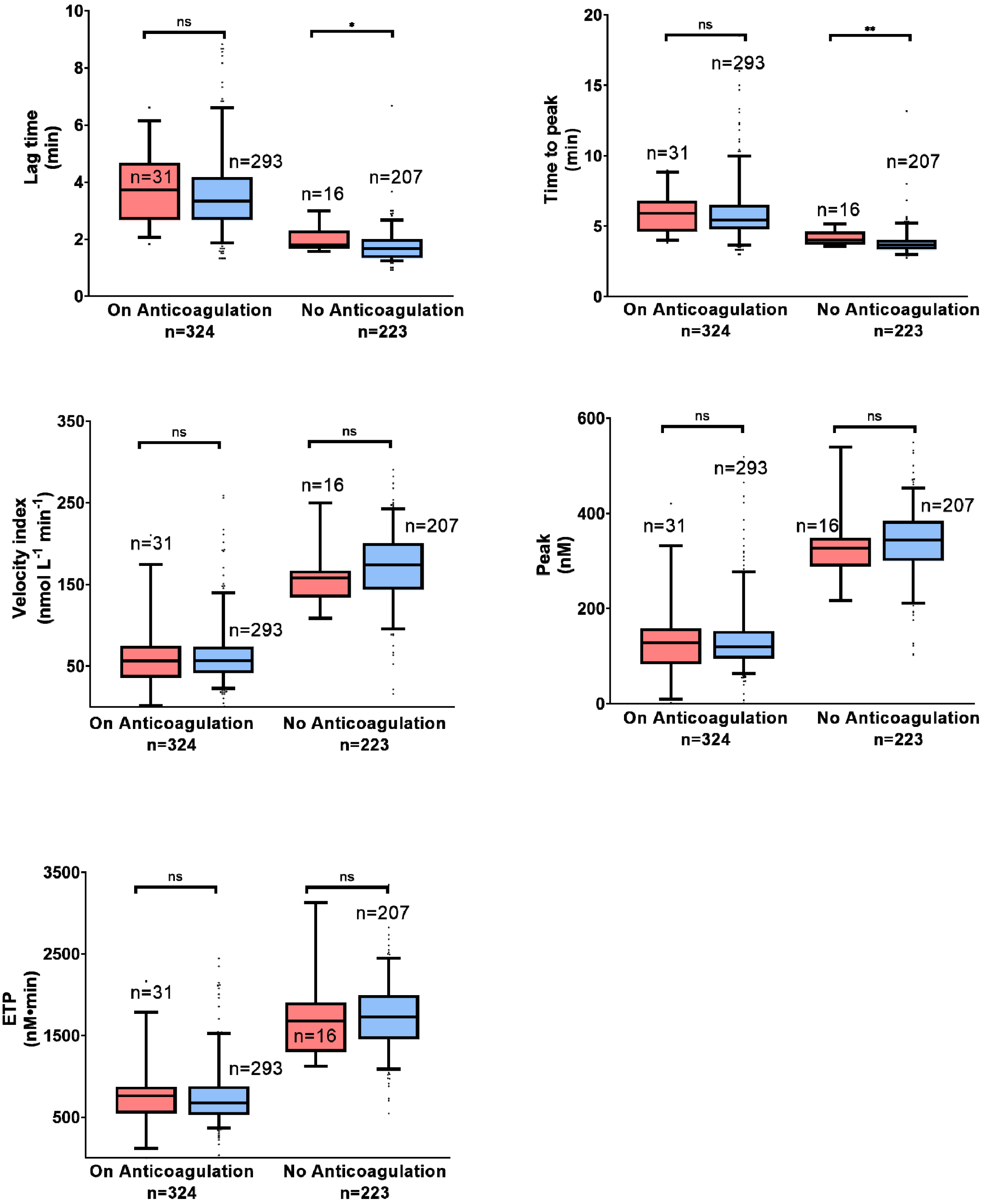

Figure S11. Thrombin generation parameters in patients under anticoagulation and not under anticoagulation after index venous thromboembolism using 13.6 pM tissue factor without activated protein C. The red boxes indicate patients who died and the blue boxes, those who did not up to 24 months following the index VTE. Box-plots of thrombin generation parameters are presented as median with interquartile range (5-95%) are indicated. Groups were compared using the Mann-Whitney U test. ETP, endogenous thrombin potential; ns, not significant; **P<0.01; *P<0.05

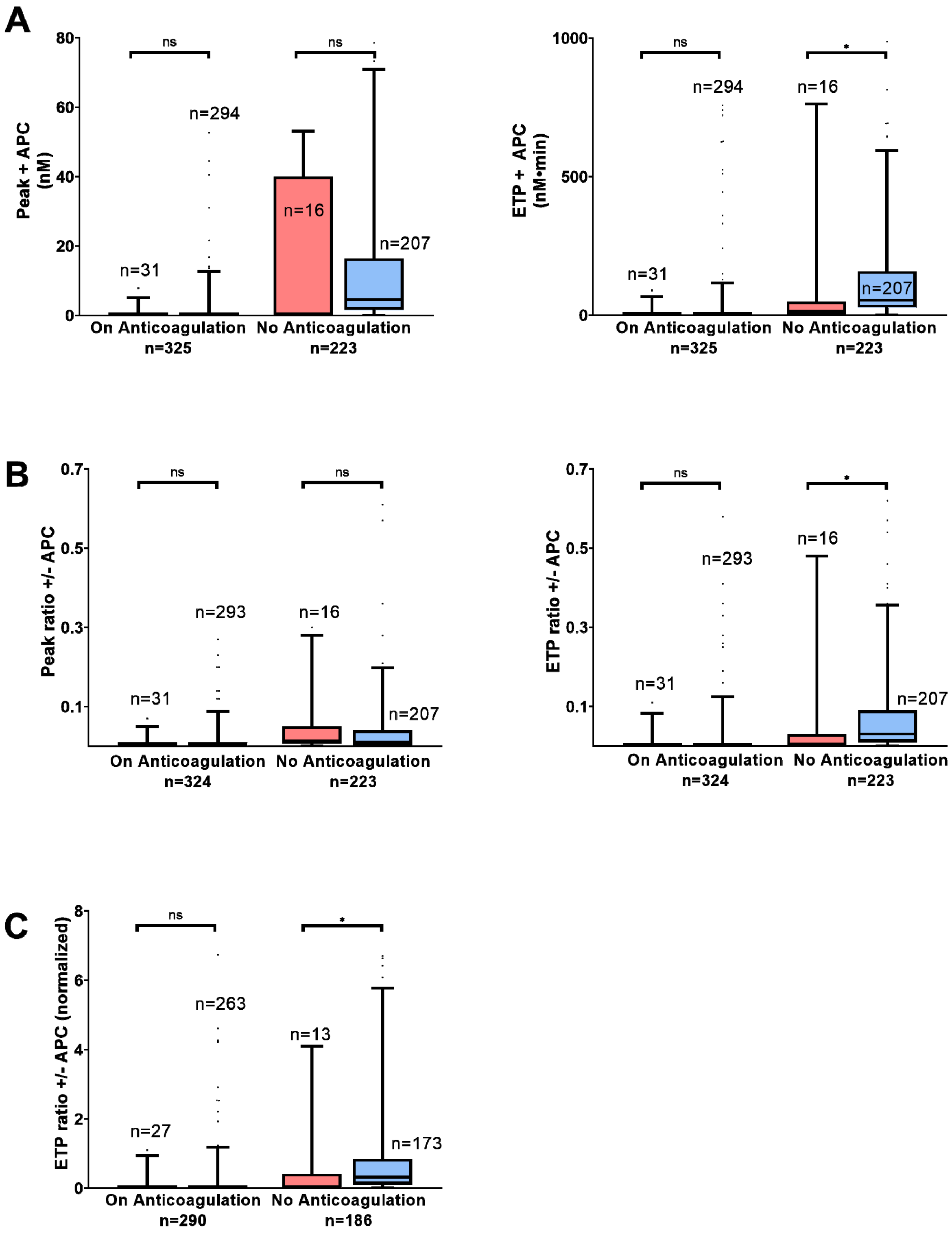

Figure S12. Peak and endogenous thrombin potential (ETP) in patients under anticoagulation and not under anticoagulation after index venous thromboembolism using 13.6 pM tissue factor with activated protein C (APC) (A), calculated ratio for endogenous thrombin potential (ETP) and peak (B) and normalized ratio (C) for peak and ETP with and without APC. The red boxes indicate patients who died and the blue boxes, those who did not up to 24 months following the index VTE. Box-plots of thrombin generation parameters are presented as median with interquartile range (5-95%) are indicated. Groups were compared using the Mann-Whitney U test. ns, not significant; *P<0.05

References

1. Agnelli G, Prandoni P, Becattini C, Silingardi M, Taliani MR, Miccio M, Imberti D, Poggio R, Ageno W, Pogliani E *et al*: **Extended oral anticoagulant therapy after a first episode of pulmonary embolism**. *Annals of internal medicine* 2003, **139**(1):19-25.
2. Boutitie F, Pinede L, Schulman S, Agnelli G, Raskob G, Julian J, Hirsh J, Kearon C: **Influence of preceding length of anticoagulant treatment and initial presentation of venous thromboembolism on risk of recurrence after stopping treatment: analysis of individual participants' data from seven trials**. *Bmj* 2011, **342**:d3036.
3. Christiansen SC, Lijfering WM, Helmerhorst FM, Rosendaal FR, Cannegieter SC: **Sex difference in risk of recurrent venous thrombosis and the risk profile for a second event**. *Journal of thrombosis and haemostasis : JTH* 2010, **8**(10):2159-2168.
4. Hansson PO, Sorbo J, Eriksson H: **Recurrent venous thromboembolism after deep vein thrombosis: incidence and risk factors**. *Archives of internal medicine* 2000, **160**(6):769-774.
5. Heit JA, Mohr DN, Silverstein MD, Petterson TM, O'Fallon WM, Melton LJ, 3rd: **Predictors of recurrence after deep vein thrombosis and pulmonary embolism: a population-based cohort study**. *Archives of internal medicine* 2000, **160**(6):761-768.
6. Huang W, Goldberg RJ, Anderson FA, Cohen AT, Spencer FA: **Occurrence and predictors of recurrence after a first episode of acute venous thromboembolism: population-based Worcester Venous Thromboembolism Study**. *Journal of thrombosis and thrombolysis* 2016, **41**(3):525-538.
7. Insam C, Mean M, Limacher A, Angelillo-Scherrer A, Aschwanden M, Banyai M, Beer JH, Bounameaux H, Egloff M, Frauchiger B *et al*: **Anticoagulation Management Practices and Outcomes in Elderly Patients with Acute Venous Thromboembolism: A Clinical Research Study**. *PloS one* 2016, **11**(2):e0148348.
8. Lopez-Jimenez L, Montero M, Gonzalez-Fajardo JA, Arcelus JI, Suarez C, Lobo JL, Monreal M, Investigators R: **Venous thromboembolism in very elderly patients: findings from a prospective registry (RIETE)**. *Haematologica* 2006, **91**(8):1046-1051.
9. Prandoni P, Lensing AW, Piccioli A, Bernardi E, Simioni P, Girolami B, Marchiori A, Sabbion P, Prins MH, Noventa F *et al*: **Recurrent venous thromboembolism and bleeding complications during anticoagulant treatment in patients with cancer and venous thrombosis**. *Blood* 2002, **100**(10):3484-3488.
10. Wattanakit K, Cushman M, Stehman-Breen C, Heckbert SR, Folsom AR: **Chronic kidney disease increases risk for venous thromboembolism**. *Journal of the American Society of Nephrology : JASN* 2008, **19**(1):135-140.
11. Beyth RJ, Quinn LM, Landefeld CS: **Prospective evaluation of an index for predicting the risk of major bleeding in outpatients treated with warfarin**. *The American journal of medicine* 1998, **105**(2):91-99.
12. Fang MC, Go AS, Chang Y, Borowsky LH, Pomernacki NK, Udaltsova N, Singer DE: **A new risk scheme to predict warfarin-associated hemorrhage: The ATRIA (Anticoagulation and Risk Factors in Atrial Fibrillation) Study**. *Journal of the American College of Cardiology* 2011, **58**(4):395-401.
13. Gage BF, Yan Y, Milligan PE, Waterman AD, Culverhouse R, Rich MW, Radford MJ: **Clinical classification schemes for predicting hemorrhage: results from the National Registry of Atrial Fibrillation (NRAF)**. *American heart journal* 2006, **151**(3):713-719.
14. Hutten BA, Prins MH, Gent M, Ginsberg J, Tijssen JG, Buller HR: **Incidence of recurrent thromboembolic and bleeding complications among patients with venous thromboembolism in relation to both malignancy and achieved international normalized ratio: a retrospective analysis**. *Journal of clinical oncology : official journal of the American Society of Clinical Oncology* 2000, **18**(17):3078-3083.
15. Kuijer PM, Hutten BA, Prins MH, Buller HR: **Prediction of the risk of bleeding during anticoagulant treatment for venous thromboembolism**. *Archives of internal medicine* 1999, **159**(5):457-460.
16. Landefeld CS, Goldman L: **Major bleeding in outpatients treated with warfarin: incidence and prediction by factors known at the start of outpatient therapy**. *The American journal of medicine* 1989, **87**(2):144-152.
17. Lip GY, Frison L, Halperin JL, Lane DA: **Comparative validation of a novel risk score for predicting bleeding risk in anticoagulated patients with atrial fibrillation: the HAS-BLED (Hypertension, Abnormal Renal/Liver Function, Stroke, Bleeding History or**

Predisposition, Labile INR, Elderly, Drugs/Alcohol Concomitantly) score. *Journal of the American College of Cardiology* 2011, **57**(2):173-180.
18. Nieto JA, Bruscas MJ, Ruiz-Ribo D, Trujillo-Santos J, Valle R, Ruiz-Gimenez N, Monreal M, Investigators R: **Acute venous thromboembolism in patients with recent major bleeding. The influence of the site of bleeding and the time elapsed on outcome**. *Journal of thrombosis and haemostasis : JTH* 2006, **4**(11):2367-2372.
19. Olesen JB, Lip GY, Hansen PR, Lindhardsen J, Ahlehoff O, Andersson C, Weeke P, Hansen ML, Gislason GH, Torp-Pedersen C: **Bleeding risk in 'real world' patients with atrial fibrillation: comparison of two established bleeding prediction schemes in a nationwide cohort**. *Journal of thrombosis and haemostasis : JTH* 2011, **9**(8):1460-1467.
20. Pisters R, Lane DA, Nieuwlaat R, de Vos CB, Crijns HJ, Lip GY: **A novel user-friendly score (HAS-BLED) to assess 1-year risk of major bleeding in patients with atrial fibrillation: the Euro Heart Survey**. *Chest* 2010, **138**(5):1093-1100.
21. Prandoni P, Lensing AW, Cogo A, Cuppini S, Villalta S, Carta M, Cattelan AM, Polistena P, Bernardi E, Prins MH: **The long-term clinical course of acute deep venous thrombosis**. *Annals of internal medicine* 1996, **125**(1):1-7.
22. Ruiz-Gimenez N, Suarez C, Gonzalez R, Nieto JA, Todoli JA, Samperiz AL, Monreal M, Investigators R: **Predictive variables for major bleeding events in patients presenting with documented acute venous thromboembolism. Findings from the RIETE Registry**. *Thrombosis and haemostasis* 2008, **100**(1):26-31.
23. Shireman TI, Mahnken JD, Howard PA, Kresowik TF, Hou Q, Ellerbeck EF: **Development of a contemporary bleeding risk model for elderly warfarin recipients**. *Chest* 2006, **130**(5):1390-1396.
24. Torn M, Bollen WL, van der Meer FJ, van der Wall EE, Rosendaal FR: **Risks of oral anticoagulant therapy with increasing age**. *Archives of internal medicine* 2005, **165**(13):1527-1532.
25. van der Meer FJ, Rosendaal FR, Vandenbroucke JP, Briet E: **Bleeding complications in oral anticoagulant therapy. An analysis of risk factors**. *Archives of internal medicine* 1993, **153**(13):1557-1562.
26. White RH, Beyth RJ, Zhou H, Romano PS: **Major bleeding after hospitalization for deep-venous thrombosis**. *The American journal of medicine* 1999, **107**(5):414-424.
27. Gussoni G, Frasson S, La Regina M, Di Micco P, Monreal M, Investigators R: **Three-month mortality rate and clinical predictors in patients with venous thromboembolism and cancer. Findings from the RIETE registry**. *Thrombosis research* 2013, **131**(1):24-30.
